# Dose-dependent activation of the Hippo pathway by Type I and Type III interferons suppresses tissue repair by human bronchial epithelial cells

**DOI:** 10.1371/journal.pbio.3003615

**Published:** 2026-01-26

**Authors:** Krupakar V. Subramaniam, Hui Jing Lim, Bao Wang, Valia T. Mihaylova, Evrett N. Thompson, Diane S. Krause, Ellen F. Foxman

**Affiliations:** 1 Department of Laboratory Medicine, Yale School of Medicine, New Haven, Connecticut, United States of America; 2 Department of Immunobiology, Yale School of Medicine, New Haven, Connecticut, United States of America; 3 Department of Cell Biology, Yale School of Medicine, New Haven, Connecticut, United States of America; 4 Yale Stem Cell Center, Yale School of Medicine, New Haven, Connecticut, United States of America; Cleveland Clinic Florida, UNITED STATES OF AMERICA

## Abstract

Interferons (IFNs) are potent antiviral cytokines that are rapidly activated when infected cells sense a virus, but continued IFN production following acute infection is linked to impaired recovery. IFNs protect against infection by inducing a suite of antiviral effectors in IFN receptor-expressing cells via JAK/STAT signaling. However, how IFNs curtail tissue repair is not fully understood. Here, we studied the effects of Type III IFNs (IFNλ1 and IFNλ2) and Type I IFN (IFNβ) on tissue repair functions of human bronchial epithelial cells (HBEC). We show that both Type III IFNs and IFNβ reduce bronchial epithelial cell migration and proliferation through a common upstream mechanism: activation of LATS1, a kinase best known for limiting organ growth as part of the Hippo signaling pathway. Mechanistically, Type III IFN or IFNβ curtailed wound healing by triggering phosphorylation of LATS1 via JAK activity, bypassing activation of MST1/2, the canonical activator of LATS1 in the Hippo pathway. Further experiments showed that distinct signaling pathways lead to LATS1 and STAT1 phosphorylation downstream of IFN receptor signaling. STAT1 was dispensable for IFN-mediated LATS1 phosphorylation and suppression of tissue repair, although as expected STAT1 was required for IFN-mediated protection from rhinovirus or influenza infection. Dose–response curve experiments revealed that higher concentrations of IFN were required to trigger LATS1 phosphorylation compared to STAT1 phosphorylation. Consistently, during rhinovirus or influenza virus infection of organotypic HBEC cultures, we observed phosphorylation of both LATS1 and STAT1, but with different kinetics, with LATS1 activation showing earlier resolution compared to STAT1 activation. These results provide a conceptual framework for understanding how IFN receptor signaling differentially controls epithelial functions required for tissue repair and antiviral defense, and inform efforts to target pathological effects of IFNs following viral infection and in other high IFN states.

## Abstract 2

Interferons (IFNs) initiate antiviral defense when infected cells sense a virus, protecting against infection via JAK/STAT signaling, but can hinder recovery by impairing tissue repair by an unknown mechanism. This study shows that at high concentrations, Type I and III IFN suppress bronchial cell migration and proliferation during tissue repair via JAK-driven LATS1 activation independent of STAT1, separating repair inhibition from antiviral defense.

## Introduction

Recovery from an infection requires a sequence of defensive programs: first, elimination of the pathogen, followed by repair and regeneration of damaged tissue. During acute viral infection, one of the most potent defenses is the interferon (IFN) response, in which viral recognition triggers secretion of IFNs by infected cells, which act on both infected and bystander cells to create an inhospitable environment for viral replication [1]. Regulated progression through defense and repair programs is expected to be crucial for optimal host defense and recovery. However, the signaling mechanism(s) that coordinate the progression from antiviral defense to tissue repair are not fully understood.

Several lines of evidence indicate that, in addition to their well-known role in antiviral defense, IFNs also suppress tissue repair. Prolonged IFN activity is associated with delayed recovery following acute viral respiratory infections for both influenza and SARS-CoV-2 in humans and animal models [2–4]. In a mouse model of influenza virus infection, knockout of Type I or Type III IFN receptors on epithelial cells facilitated recovery of mouse lungs post-infection [5]. Type I IFNs are produced by many cell types and can act on most cells in the body, whereas Type III IFNs are largely produced in barrier tissues such as the respiratory mucosa and act only on barrier epithelial cells and a few other cell types [6]. Both IFN types activate JAK/STAT signaling pathways through their respective receptors, inducing expression of a large family of antiviral genes known as interferon-stimulated genes (ISGs) [7]. The effects of IFN receptor signaling on tissue repair are not as well studied. In the mouse influenza infection model, both Type I and Type III IFNs were shown to attenuate lung repair through activation of p53 in respiratory epithelial cells, reducing cell proliferation [5]. Interestingly, cell migration, another key process for epithelial repair, is also reported to be inhibited by Type I IFNs in some cell types [8–10], suggesting that IFN-mediated suppression of distinct tissue repair processes like migration and proliferation may occur through common upstream signals.

A promising candidate is the Hippo signaling pathway, which regulates Yes-associated protein (YAP) and transcriptional co-activator with PDZ-binding motif (TAZ) to promote diverse tissue growth and repair activities including proliferation and migration in response to subconfluence [11]. YAP/TAZ are regulated by the Hippo signaling cascade first discovered in fruit flies [12]. Hippo (mammalian homologue MST1/2) is a serine/threonine protein kinase activated by cell confluence to limit organ size, which is accomplished through a kinase cascade that ultimately phosphorylates and activates LATS1/2, which in turn phosphorylates YAP, sequestering it in the cytoplasm and targeting it for degradation. Conversely, when MST1/2 and LATS1/2 are inactive, YAP concentration increases, and YAP translocates to the nucleus to induce expression of its target genes, promoting tissue growth. In addition to cell confluence and mechanical cues, other pathways have been shown to intersect with this signaling cascade, including antiviral signaling. A prior study showed that in some cell types, Type I IFN receptor signaling can induce LATS1 phosphorylation, and in turn, activated LATS1 can reinforce the antiviral response by promoting maximal activation of STAT1, a key signaling molecule activated by IFN receptor to induce expression of ISGs [13]. In that study, Type II and Type III IFNs did not activate LATS1; however, the experiment was done in a fibrosarcoma cell line, which may contain different signaling circuits than primary human cells. Conversely, YAP activity has been shown to suppress antiviral signaling by intracellular innate immune sensors for viral RNA and DNA that initiate the IFN response [14–16].

Motivated by prior evidence for interactions between these signaling pathways and recent evidence that IFNs can inhibit lung repair following acute viral infection [3,17], here we explored whether IFN signaling suppresses human respiratory epithelial cell repair via effects on LATS1 and YAP. We tested both Type I and Type III IFNs but focused on Type III IFN (IFNλ), the major IFN type produced by respiratory epithelial cells in response to viral infection [18,19]. We found that both Type I and Type III IFNs curtail tissue repair in epithelial cells and fibroblasts by co-opting the downstream components of the Hippo signaling pathway, and that this function requires JAK1, but not MST1/2 or STAT1 activity. We also show LATS1 and STAT1 phosphorylation have distinct dose–response curves following IFN receptor activation, with LATS1 phosphorylation requiring higher concentrations of IFN and resolving more quickly following acute viral infection. These results reveal a unifying mechanism for how IFN receptor signaling limits epithelial proliferation and migration and show that IFN receptor engagement initiates distinct dose-dependent signaling events regulating antiviral defense and tissue repair.

## Results

### Both Type III and Type I IFN slow wound healing of primary human bronchial epithelial cells (HBEC)

**Fig 1:**
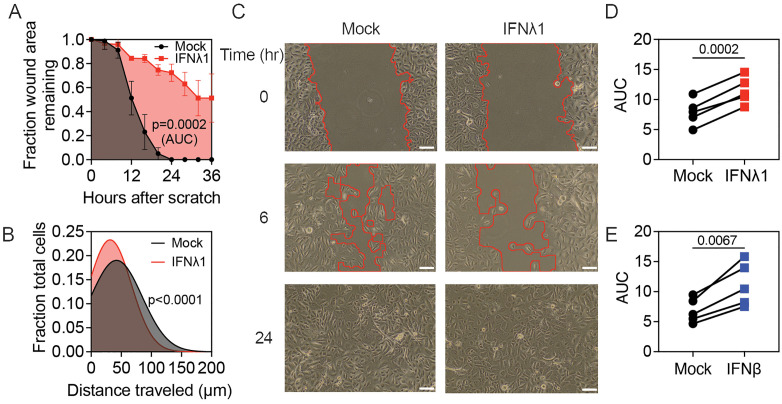
Type III or Type I IFN reduces wound healing in human bronchial epithelial cells. **(A, B)** HBEC were grown to confluence in 10 mm glass plates, then wounded with a standardized linear scratch (300 µm). **(A)** Fraction of wound area remaining with and without IFNλ1 pretreatment (50 ng/mL) was calculated using time-lapse microscopy. Mean and SEM of four replicates per condition are shown, with area under the curve (AUC) compared using Welch’s *t* test. **(B)** Distribution of distance traveled for 300 cells per condition, fit to a histogram. Means of raw data were compared using Welch’s *t* test. **(C–E)** HBEC were plated in polystyrene 24-well plates and grown to confluence for 72 hours, followed by **(C, D)** IFNλ1 (50ng/mL) or **(E)** IFNβ (5 ng/ml) pretreatment and wounding with a standardized linear scratch (600 µm). **(C)** Photomicrographs showing wound area for mock and IFNλ1-treated HBEC at 0-, 6-, and 24-hours post scratch, with edge of wound area highlighted in red. For all images, scale bar = 100 µm. **(D, E)** AUC for wound healing over 24 hours for paired mock- and **(D)** IFNλ1-treated or **(E)** IFNβ-treated cultures from five independent experiments. Means compared using paired *t* test. The data underlying this figure can be found in S1 Data and at https://doi.org/10.17632/6rvkfsy2n8.1.

Both Type III and Type I IFNs are made by airway epithelial cells responding to viral infection, but Type III IFN predominates, therefore we studied how both IFN types affect epithelial repair with an emphasis on Type III IFNs. To determine the effect of IFNs on wound healing in primary HBEC, we used a scratch test, a classical model used to evaluate wound healing by different cell types [20,21]. First, we observed the kinetics of wound healing and the effect of IFNλ1 exposure using time-lapse microscopy. We introduced a standardized 300 µm linear scratch in HBEC monolayers cultured to confluence on a 10 mm collagen-coated glass dish compatible with the Vivaview time-lapse microscopy system. Mock-treated cells healed the wound by 24 hours, but IFNλ1-pretreated cells showed a significant defect, healing to only 51% of the initial wound area by 36 hours after the scratch was introduced (Fig 1A).

Prior work showed that Type I and III IFNs reduce lung repair by inhibiting epithelial cell proliferation [5], but it was unclear whether this was responsible for reduced wound healing in the scratch test model since primary HBEC proliferate at a slow rate relative to the duration of the assay. To assess this, we measured proliferation of subconfluent HBEC with and without IFNλ1. For mock-treated cells, the average cell number increased 1.3-fold in 48 hours. This rate was not significantly reduced by IFNλ1 treatment, although there was a trend towards a slight reduction in cell number (S1 Fig). This result suggests that additional mechanisms contribute to the effect of IFNλ1 on HBEC wound healing in the scratch test model. We next assessed the effect of IFNλ1 on cell migration. Using time-lapse microscopy and trajectory analysis, we found that mock-treated cells traveled a significantly greater distance than IFNλ1-pretreated cells following introduction of a scratch: for 300 cells tracked per condition, the average distances traveled were 67 and 52 µm for mock- and IFNλ1-pretreated cells, respectively (Fig 1B). Time-lapse microscopy showed that cells in the IFNλ1-pretreated group tended to remain near the edge of the scratch, whereas cells from the mock-pretreated group more frequently migrated across the wound (S1 and S2 Videos). Together, these data indicate that reduced HBEC wound healing in the presence of IFNλ1 is likely due primarily to an effect on cell migration rather than cell proliferation over the time scale of this assay.

Next, we switched to a higher throughput assay for wound healing, in which HBEC were cultured to confluence in 24-well collagen-coated plates and micrographs were taken at 6-hour intervals to quantify wound area remaining (Fig 1C). This format enabled us to efficiently compare wound healing when using multiple conditions and experimental replicates. In this model, both mock and IFNλ1-treated cells healed a standardized 600 µm scratch within 24 hours, but similar to what we observed by time lapse microscopy, the rate of wound healing was consistently reduced in IFNλ1-pretreated compared to mock-treated cultures (Fig 1D). We quantified rate of wound healing as the area under the curve (AUC) of a graph of wound area (y-axis) over time (x-axis). We also tested IFNβ, a Type I IFN, using this model. Like IFNλ1, IFNβ inhibited wound healing (Fig 1E), consistent with prior work in a mouse model showing that epithelial Type I and Type III IFN receptors participate in reduced lung repair following influenza infection [5].

SiRNA knockdown (KD) of *IFNLR1*, the limiting subunit of the Type III IFN receptor, abrogated the effect of IFNλ1 on wound healing, demonstrating the requirement of IFN receptor signaling for this effect (S2 Fig).

### Type III and Type I IFN reduce YAP nuclear activity in HBEC

**Fig 2:**
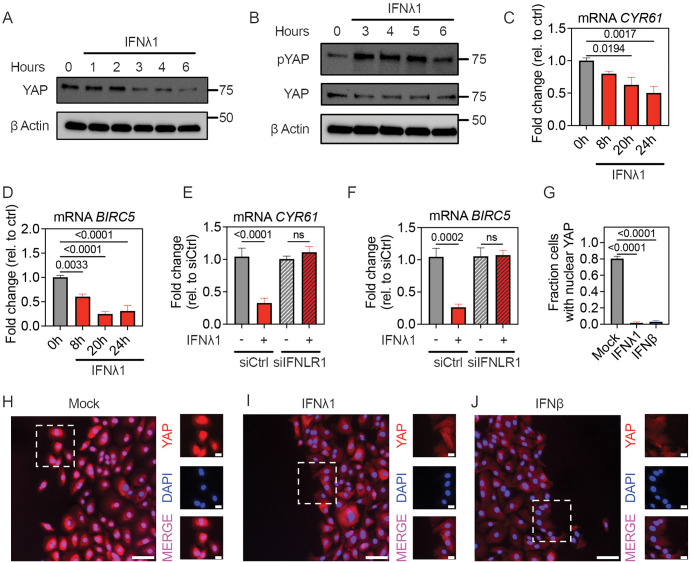
Exposure to Type III or Type I IFN inhibits YAP nuclear activity. **(A, B)** Subconfluent HBEC were treated with IFNλ1 (50 ng/ml) and cell lysates were collected at indicated time points for western blot. **(A)** Total YAP at hourly intervals after treatment. **(B)** pYAP Ser^397^ and total YAP after IFNλ1 treatment at specified times. **(C–F)** Subconfluent HBEC were treated with IFNλ1 for specified times, then RNA was isolated, followed by RT-qPCR for YAP target genes *CYR61* or *BIRC5*. Graphs show mean and SEM of six replicates per condition, with means compared using Ordinary One-Way ANOVA test relative to expression level in untreated cells. **(C, D)** Effect of IFNλ1 treatment on YAP target gene expression at indicated time points. **(E, F)** Effect of IFNλ1 treatment on YAP target gene expression 24 hours post-IFNλ1 exposure in HBEC transfected with siCtrl and siIFNLR1. **(G–J)** HBEC treated with medium only, IFNλ1 (50 ng/mL) or IFNβ (5 ng/mL) were fixed four hours after wounding with a standardized linear scratch (600 µm) and stained for YAP and DAPI. **(G)** Fraction of cells within 100 µm of the scratch with YAP (red)-DAPI (blue; nucleus) co-localization. Mean and SEM of eight high-power fields per condition are shown and compared using Ordinary One-Way ANOVA test. **(H–J)** Insets represent field of view captured by dashed white box, showing merged channel (bottom) or individual YAP (red) and DAPI (blue) channels. Scale bar = 100 µm for large images and 20 µm for insets. The data underlying this figure can be found in S1 Data, S1 Raw Images, and at https://doi.org/10.17632/6rvkfsy2n8.1.

To probe possible mechanisms for the inhibitory effects of IFNs on HBEC wound healing, we first evaluated the effect of IFNs on YAP activity. We studied subconfluent HBEC, a condition in which YAP is expected to be active in the nucleus. Using western blot, we observed a decrease in total cellular YAP and an increase in phosphorylation of YAP at Ser^397^, which targets the protein for proteasomal degradation [22], following IFNλ1 treatment (Fig 2A and 2B). IFNλ1 also led to a time-dependent decrease in expression of YAP target genes *CYR61* and *BIRC5* [23] (Fig 2C and 2D). Knocking down *IFNLR1* rescued the expression of *CYR61* and *BIRC5* after IFNλ1 treatment (Fig 2E and 2F), confirming that IFNλ1-Type III IFN receptor signaling was responsible for disrupting YAP nuclear activity. We next investigated the effect of IFNλ1 on YAP localization in confluent HBEC cultures four hours after the introduction of a linear scratch used for the wound healing assay. In untreated cultures, YAP was largely localized to the nucleus at four hours post-wounding in cells within 100 µm of the scratch, but in cultures pretreated with IFNλ1, YAP was almost entirely localized to the cytoplasm (Fig 2G–2I). Similar to what we observed with IFNλ1, treating subconfluent HBEC with IFNβ also reduced expression of YAP target genes (S3 Fig) and nuclear localization of YAP post-wounding (Fig 2G and 2J).

We next evaluated the effect of IFNλ1 and IFNβ on expression of genes that promote migration. In addition to *CYR61*, expression of YAP-dependent migration genes *TRIO* and *CTGF* [24,25] decreased after IFNλ1 and IFNβ treatment (S4A and S4B Fig). Conversely, expression of *EGR3* and *VIM*, genes previously found to regulate cell migration in a YAP-independent manner [26,27], did not change after IFNλ1 and IFNβ treatment (S4C and S4D Fig), supporting the idea that IFNs disrupt HBEC migration in a YAP-dependent manner.

Because YAP is rapidly phosphorylated and degraded after IFNλ1 exposure, we also evaluated whether IFNλ1 pretreatment used in our initial protocol (Fig 1) was necessary for inhibition of wound healing. Treating HBEC with IFNλ1 at the time of the scratch was sufficient to inhibit wound healing, though pretreatment enhanced the effect (S5 Fig).

Together, these results show that HBEC exposure to Type III or Type I IFN reduces total YAP, nuclear YAP localization, and the activity of YAP as a transcriptional co-regulator of target genes associated with epithelial repair.

### Inhibition of wound healing by Type III and Type I IFN is LATS1/2-dependent

**Fig 3:**
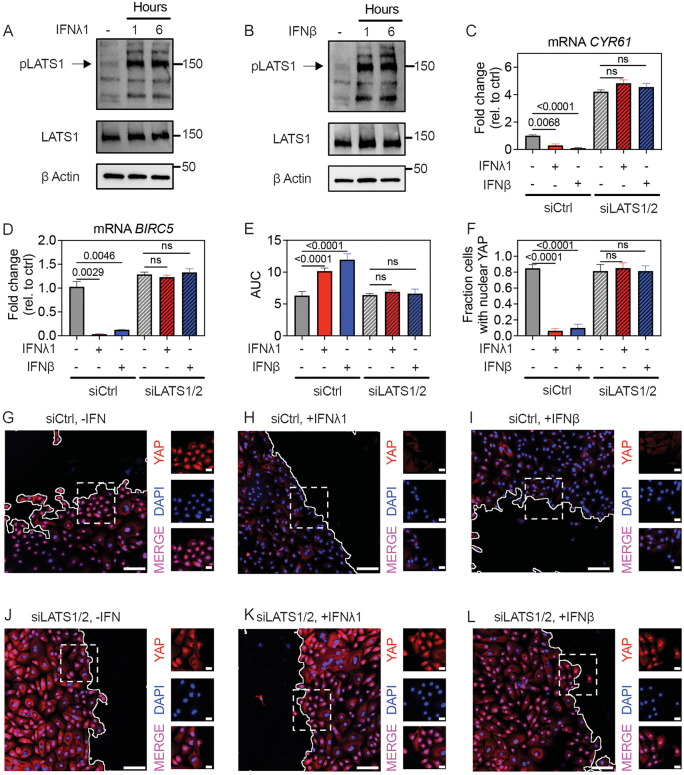
Exposure to Type III or Type I IFN blocks tissue repair in a LATS-dependent manner. **(A, B)** Subconfluent HBEC were treated with **(A)** IFNλ1 (50 ng/mL) or **(B)** IFNβ (5 ng/mL) for specified times, followed by western blot on cell lysates for pLATS1 Ser^909^ and total LATS1. **(C, D)** HBEC were transfected with control or LATS1/2 siRNA, then treated with IFNλ1 and IFNβ for 24 hours, followed by RNA isolation and RT-qPCR for *CYR61* or *BIRC5*. Graphs show mean and SEM of six replicates per condition, with means compared using Brown–Forsythe and Welch ANOVA tests. **(E, F)** siCtrl or siLATS1/2 HBEC were grown to confluence, followed by IFNλ1 or IFNβ treatment and wounding with a standardized linear scratch (600 µm). AUCs quantifying fraction of wound area remaining over 24 hours are shown as mean and SEM of six replicates per condition and compared using Ordinary One-Way ANOVA test. **(F–L)** YAP (red) and DAPI (blue) staining of siCtrl or siLATS1/2 HBEC treated with IFNλ1 or IFNβ, four hours after standardized linear scratch (600 µm). **(F)** Fraction of cells within 100µm of the scratch with YAP-DAPI co-localization. Mean and SEM of six high-power fields per condition are shown and compared using Ordinary One-Way ANOVA test. **(G–L)** Insets represent field of view captured by dashed white box, showing merged channel (bottom) or individual YAP (red) and DAPI (blue) channels. Scale bar = 100 µm for large images and 20 µm for insets. The data underlying this figure can be found in S1 Data, S1 Raw Images, and at https://doi.org/10.17632/6rvkfsy2n8.1.

We next asked whether LATS1/2, a negative regulator upstream of YAP in the Hippo pathway, plays a role in reducing YAP nuclear activity in response to IFN. Phosphorylation of LATS1 at Ser^909^ increases its kinase activity and promotes subsequent autophosphorylation [28]. By Western Blot, both IFNλ1 and IFNβ increased detection of pLATS1 Ser^909^ (Fig 3A and 3B). Next, we examined the effect of *LATS1/2* siRNA KD on nuclear YAP nuclear localization and activity and effect on wound healing. In subconfluent HBEC, *LATS1/2* KD increased baseline expression of YAP target genes and abrogated their suppression by IFNλ1 and IFNβ (Figs 3C, 3D, and S6). *LATS1/2* KD also rescued the effect of IFNλ1 or IFNβ on wound healing and nuclear localization of YAP in the scratch assay model (Fig 3E–3L). Without IFN treatment, the fraction of cells with nuclear YAP was equally high (~80% in both control and LATS1/2 KD cells) and the rate of wound healing was equivalent (Fig 3E, 3F, 3G, and 3J). These observations suggest that following wounding, YAP is maximally activated such that LATS1/2 only curtails YAP activity when HBEC are co-exposed to IFNs. Taken together, these data support a model in which LATS kinases mediate the inhibition of HBEC wound healing by IFNλ1 and IFNβ.

In the classical Hippo pathway, MST1/2 kinases become activated by phosphorylation in response to cellular confluence and other environmental cues, and then phosphorylate and activate LATS1/2 to curtail tissue growth and repair [11]. However, in our experiments neither IFNλ1 nor IFNβ caused phosphorylation of MST1 in HBEC, while HBEC treated with staurosporine (STS) as a positive control did show MST1 phosphorylation as expected [29] (S7A Fig). To further evaluate whether MST1/2 are involved in phosphorylation of LATS1 downstream of IFN receptor signaling, we utilized the well-characterized MST1/2 kinase inhibitor XMU-MP-1 (XMU) [30], which inhibited MST1/2 activity in HBEC at 10µM with minimal cell toxicity (S7B Fig). While pretreatment of HBEC at this dose abrogated LATS1 phosphorylation in response to STS, XMU had no effect on LATS1 phosphorylation in response to IFNλ1 (S7C Fig). XMU also had no effect on IFNλ1 or IFNβ-mediated suppression of YAP target gene expression in subconfluent cells or wound healing in scratched monolayers (S7D–S7F Fig). These results demonstrate that MST1/2 are not required for IFN-mediated activation of LATS1/2 kinases or for IFN-mediated suppression of tissue repair.

### Exposure to Type III or Type I IFN suppresses bronchial epithelial cell proliferation via LATS1/2 and P53

**Fig 4:**
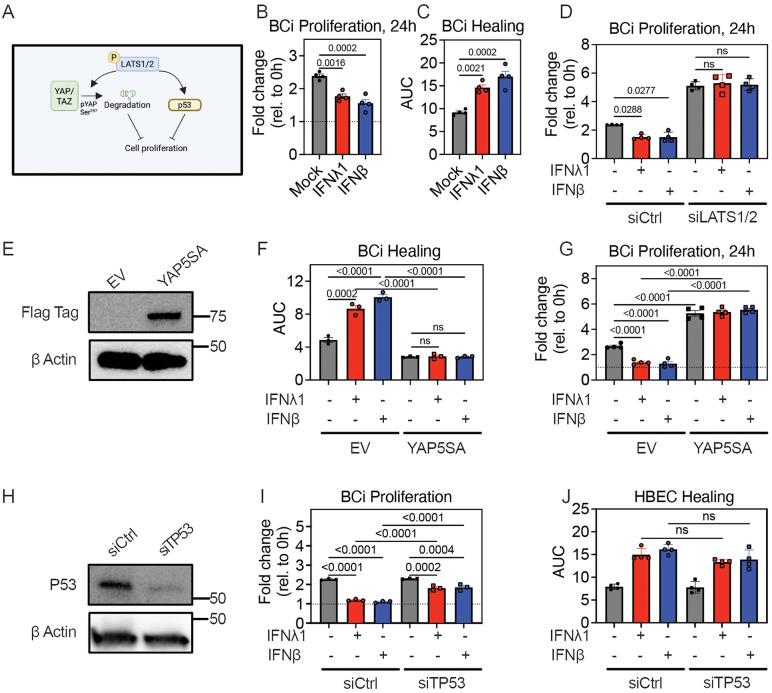
Exposure to Type III or Type I IFN inhibits proliferation of bronchial epithelial cells via effects of LATS1/2 and P53. **(A)** Model for relationship between LATS1/2, YAP, and P53 in inhibiting cell proliferation. **(B)** Subconfluent BCi were treated with IFNλ1 or IFNβ for 24 hours. Fold change in cell count from 0 to 24 hours is shown, with dashed line at 1 representing starting cell number. Graph shows mean and SEM of four replicates per condition, with means compared using Ordinary One-Way ANOVA test. **(C)** BCi were grown to confluence, followed by IFNλ1 or IFNβ pretreatment and wounding with a standardized linear scratch (600 µm). Fraction of wound area remaining was quantified over time to calculate AUC. Mean and SEM of four replicates per condition are shown, with AUCs compared using Ordinary One-Way ANOVA test. (D) siCtrl and siLATS1/2-transfected BCi were treated with IFNλ1 or IFNβ for 24 hours. Fold change in cell count from 0 to 24 hours is shown, with dashed line at 1 representing starting cell number. Graph shows mean and SEM of four replicates per condition, with means compared using Ordinary One-Way ANOVA test. **(E–G)** BCi were transduced with an empty vector (EV) or YAP5SA. **(E)** western blot on EV and YAP5SA BCi lysates for Flag-tag. **(F)** EV and YAP5SA BCi were grown to confluence, followed by IFNλ1 or IFNβ treatment and wounding with a standardized linear scratch (600 µm). Fraction of wound area remaining was quantified. Graph shows mean and SEM of three replicates per condition, with means compared using Ordinary One-Way ANOVA test. **(G)** Subconfluent EV and YAP5SA BCi were treated with IFNλ1 or IFNβ for 24 hours. Fold change in cell count from 0 to 24 hours is shown, with dashed line at 1 representing starting cell number. Graphs show mean and SEM of four replicates per condition, with means compared using Ordinary One-Way ANOVA test. **(H)** western blot for p53 on cell lysates of siCtrl and siTP53-transfected HBEC four days after transfection. **(I)** siCtrl and siTP53 BCi were treated with IFNλ1 or IFNβ for 24 hours. Fold change in cell count from 0 to 24 hours is shown, with dashed line at 1 representing starting cell number. Graphs show mean and SEM of three replicates per condition, with means compared using Ordinary One-Way ANOVA test. **(J)** siCtrl and siTP53 HBEC were grown to confluence, followed by IFNλ1 (red) or IFNβ (blue) treatment and wounding with a standardized linear scratch (600 µm). Fraction of wound area remaining was quantified over time to calculate AUC. Graph shows mean and SEM of four replicates per condition, with AUCs compared using Ordinary One-Way ANOVA test. The data underlying this figure can be found in S1 Data and S1 Raw Images.

The Hippo pathway LATS1/2 kinases are known to inhibit cell proliferation, both through reducing YAP activity and through interaction with other pathways, including activation of p53 (Fig 4A) [31]. Inhibition of cell proliferation via p53 was also previously implicated in IFN-mediated inhibition of lung epithelial repair in a mouse model [5]. Therefore, we sought to test whether the LATS1/2-YAP axis also mediates effects of IFNs on bronchial epithelial proliferation and the role of p53. Because primary HBEC proliferate slowly in culture (S1 Fig), we used a faster proliferating bronchial epithelial cell line, BCi-NS1.1 (BCi cells), to investigate this question [32]. Subconfluent BCi cells proliferated faster than primary HBEC in culture, showing a 2.4-fold increase in cell number over 24 hours, and proliferation was significantly curtailed by exposure to either IFNλ1 or IFNβ (Fig 4B). In the scratch assay, BCi monolayers also healed completely from a standardized linear scratch within 24 hours in 24-well tissue culture plates, but IFNλ1 or IFNβ exposure significantly reduced the rate of wound healing (increased the AUC), with IFNλ1- and IFNβ-treated cultures showing a persistent defect at 24 hours (Fig 4C). These results show that for BCi cells, cell proliferation is curtailed by Type III or Type I IFN exposure and suggests that limiting cell proliferation contributes to the reduced rate of wound healing in the scratch test model.

To assess the role of the LATS1/2-YAP axis on IFN-mediated inhibition of cell proliferation, we performed proliferation and wound healing assays using *LATS1/2 KD* in BCi cells and BCi cells overexpressing a constitutively active form of YAP (YAP5SA) [33]. *LATS1/2* KD increased the proliferation rate and abrogated the suppressive effects of IFNλ1 or IFNβ exposure on cell proliferation (Fig 4D). Similarly, YAP5SA overexpression increased the rate of wound healing, and abrogated inhibition of wound healing by IFNλ1 or IFNβ (Fig 4E and 4F); YAP5SA overexpression also increased the basal proliferation rate and abrogated the suppressive effects of IFNλ1 or IFNβ on cell proliferation (Fig 4G).

To probe whether p53 was required for the suppressive effect of IFNs on BCi cell proliferation, we used siRNA KD. *TP53* KD partially rescued the suppressive effect of IFNλ1 or IFNβ on proliferation of BCi cells (Fig 4H and 4I), suggesting that LATS1/2 suppresses cell proliferation through both activation of p53 and suppression of YAP nuclear activity. *TP53* KD in HBEC did not significantly affect rate of wound healing or inhibition by IFNs (Fig 4J), consistent with our previous findings suggesting that HBEC wound healing in this model occurs primarily via cell migration, not cell proliferation. Together, these results support a model in which, in the setting of rapidly proliferating airway epithelial cells, IFN exposure also suppresses epithelial repair by blocking epithelial proliferation through activation of the downstream components of the Hippo pathway, including activation of LATS1/2, suppression of YAP nuclear activity, and activation of p53.

### IFN-mediated effects on wound healing require JAK activity but not STAT1

Because our results indicate that Type III or Type I mediated activation of LATS1/2 is independent of MST1, we next interrogated the role(s) of other intracellular signaling mechanisms in LATS1/2 phosphorylation. Type I and III IFN receptors signal through JAK/STAT, in which binding of IFN to its receptor causes phosphorylation of receptor-associated Janus kinases (JAKs) which then phosphorylate STAT1 and STAT2, resulting in homodimerization of STAT1 or heterodimerization of STAT1 and STAT2, followed by nuclear translocation to induce the expression of ISGs that mediate antiviral defense [34]. Prior work in a fibrosarcoma cell line showed that Type I, but not Type III, IFN receptor signaling led to JAK-dependent LATS1/2 phosphorylation, but the role of STATs and the signaling pathways involved in Type III-IFN-mediated LATS1/2 phosphorylation are not known [13].

**Fig 5:**
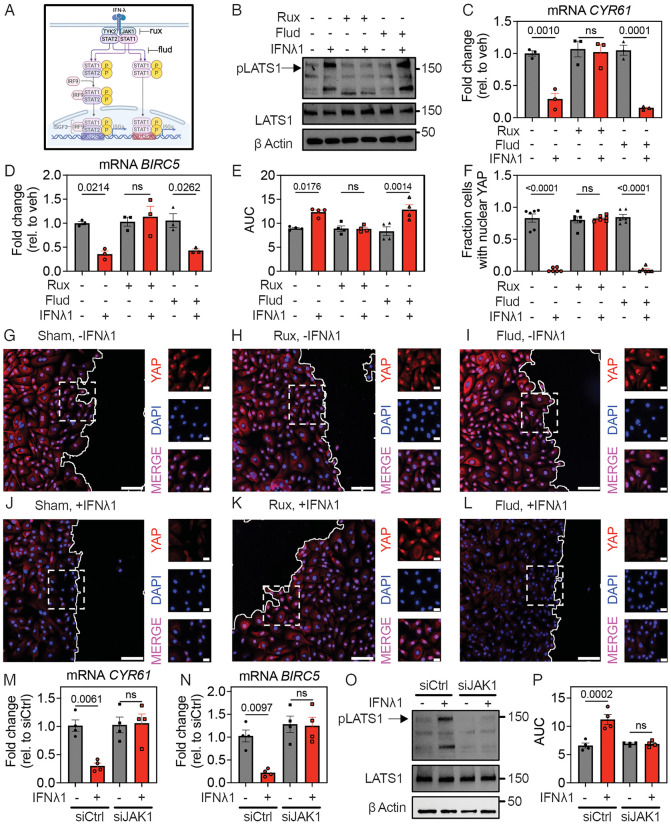
IFNλ1-mediated suppression of wound healing in bronchial epithelial cells requires JAK1 but not STAT1. **(A)** Type III IFN receptor signaling and targets of ruxolitinib (rux) and fludarabine (flud). **(B)** Subconfluent HBEC were pretreated with rux or flud prior to IFNλ1 treatment, followed by western blot on cell lysates pLATS1 Ser^909^ and total LATS1. **(C, D)** Subconfluent HBEC were pretreated with rux or flud prior to treatment with IFNλ1 for 24 hours, followed by RNA isolation and RT-qPCR for *CYR61* or *BIRC5*. Graphs show mean and SEM of three replicates per condition, with means compared using Ordinary One-Way ANOVA test. **(E)** Confluent rux- and flud-pretreated HBEC were treated with IFNλ1 followed by wounding with a standardized linear scratch (600 µm). Fraction of wound area remaining was quantified over time to calculate AUC. Graph shows mean and SEM of four replicates per condition, with means compared using Ordinary One-Way ANOVA test. **(F–L)** Rux- or flud-pretreated HBEC treated with IFNλ1 were fixed four hours after wounding with a standardized linear scratch (600 µm) and stained for YAP and DAPI. **(F)** Number of cells with nuclear YAP within 100µm of the leading edge of the scratch were quantified for each condition. Graph shows mean and SEM of six high-power fields of view per condition, with means compared using Ordinary One-Way ANOVA test. **(G–L)** For low magnification images, scale bar = 100 µm. Solid white line represents scratch border. Insets represent field of view captured by dashed white box in low magnification images and show merged channel (bottom) or individual YAP (red) and DAPI (blue) channels. For insets, scale bar = 20 µm. **(M–O)** Subconfluent siCtrl or siJAK1 HBEC were treated with IFNλ1 **(M, N)** for 24 hours, followed by RNA isolation and RT-qPCR for YAP target genes, or **(O)** for six hours, followed by western blot on cell lysates for pLATS1 Ser^909^ and total LATS1. **(P)** siCtrl or siJAK1 HBEC were grown to confluence, followed by IFNλ1 pretreatment and wounding with a standardized linear scratch (600 µm). Fraction of wound area remaining was quantified over time to calculate AUC. Graphs show mean and SEM of four replicates per condition, with means compared using Ordinary One-Way ANOVA test. The data underlying this figure can be found in S1 Data, S1 Raw Images, and at https://doi.org/10.17632/6rvkfsy2n8.1.

To probe which pathways downstream of IFN receptor signaling are necessary for LATS1 phosphorylation, we used the JAK1/2 inhibitor ruxolitinib and STAT1 inhibitor fludarabine in primary HBEC stimulated with IFNλ1 (Fig 5A) [35,36]. While both ruxolitinib and fludarabine pretreatment blocked ISG induction in response to IFNλ1, only ruxolitinib blocked LATS1 phosphorylation in response to IFNλ1 (Figs 5B and S8). Consistently, ruxolitinib, but not fludarabine, blocked IFNλ1-mediated YAP target gene expression and YAP nuclear translocation and rescued IFNλ1-mediated suppression of wound healing (Figs 5C–5L and S9).

Since fludarabine precedes STAT1 activation and ISG induction in response to IFN, it is also expected to reverse the protective effect of IFNs against viral infection. To confirm that fludarabine treatment abrogated the biological activity of STAT1, we tested the effect of this inhibitor on viral replication of two common respiratory viruses, rhinovirus A1 (RV) and a GFP-labeled influenza A virus (IAV-PR8 GFP) [37]. As expected, IFNλ1 suppressed replication of both RV and IAV in HBEC, and the protective effect of IFNλ1 was abrogated by fludarabine (S10 Fig). These results show that fludarabine blocks IFN-induced, STAT1-mediated antiviral activity.

To further evaluate the necessity of STAT1 for IFN-mediated suppression of wound healing compared to IFN-mediated antiviral defense using a genetic approach, we used primary human fibroblasts from a patient with a genetic deficiency in STAT1 (STAT1^−/−^) as previously reported [38]. We tested the effects of IFNβ on both phenotypes, since initial experiments showed that WT fibroblasts were responsive to IFNβ but not IFNλ1 (S11A and S11B Fig). IFNβ inhibited YAP target gene expression and suppressed wound healing in WT and STAT1^−/−^ fibroblasts (S11C–S11F Fig), providing genetic evidence that STAT1 signaling is not required for IFNβ-mediated suppression of wound healing. In contrast, compared to WT fibroblasts, STAT1^−/−^ fibroblasts were highly susceptible to RV or IAV infection, and STAT1 deficiency prevented IFNβ-mediated protection from these viruses (S11G–S11I Fig). These results demonstrate the biological significance of inhibition or loss of STAT1 for antiviral defense in the models used here, and together with prior results (Fig 5A–5L), further support the idea that STAT1 activity is not required for IFN-mediated suppression of wound healing.

To further evaluate the role of JAK1 in IFN-mediated LATS1 phosphorylation using an orthogonal method, we used *JAK1* siRNA KD in primary HBEC. Similar to the effect of the JAK inhibitor ruxolitinib, *JAK1* KD abrogated IFNλ1-induced YAP target gene expression, LATS1 phosphorylation, and suppression of wound healing (Figs 5M–5P and S12). These results demonstrate the necessity of JAK1 for IFNλ1-mediated inhibition of tissue repair functions.

Together, these results support a model in which there is a bifurcation of the signaling pathways downstream of Type I and III IFN receptor signaling, with JAK1 signaling leading to LATS1 phosphorylation and inhibition of tissue repair functions, or JAK/STAT1 activation leading to expression of antiviral ISGs. These results also indicate that this bifurcation occurs in both epithelial cells and fibroblasts, distinct structural cell types that participate in tissue repair.

### Respiratory virus infections activate JAK-dependent LATS1 and STAT1 phosphorylation in the differentiated human bronchial epithelium

**Fig 6:**
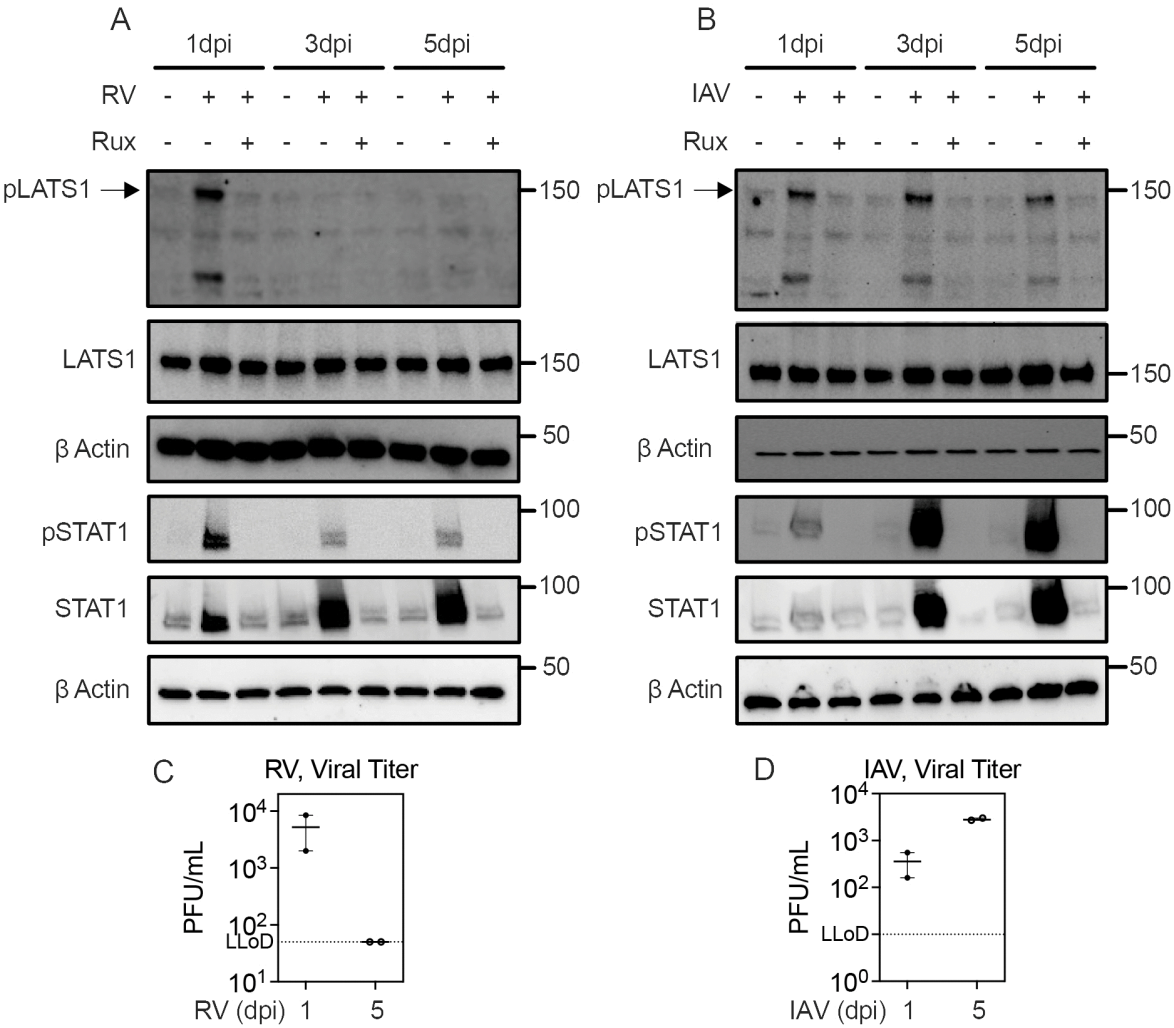
Rhinovirus or influenza A virus infection of organotypic bronchial epithelial cultures causes both LATS1 and STAT1 phosphorylation. Air–liquid interface HBEC were pretreated with ruxolitinib prior to infection with RV or IAV. **(A, B)** western blot on cell lysates from 1, 3, and 5 dpi for pLATS1 Ser^909^, total LATS1, pSTAT1 Tyr^701^, and total STAT1. Top three panels show western blot for pLATS1/LATS1/β actin and bottom three panels show separate western blot on same cell lysates for pSTAT1/STAT1/β actin. **(C, D)** RV and IAV viral load in apical wash by plaque assay at 1 and 5 dpi. Graphs show mean and SEM overlayed with two replicates. The data underlying this figure can be found in S1 Data and S1 Raw Images.

To complement our results using recombinant IFNs and to test physiological relevance, we next sought to evaluate whether LATS1 phosphorylation occurs during viral infection and its dependence on JAK1 signaling. To this end, we used the air–liquid interface (ALI) culture, an organotypic culture of the human bronchial epithelium in which primary HBEC are cultured with the apical surface exposed to air for 4 weeks, over which time they differentiate into a tissue which recapitulates many features of the human airway mucosa, including showing beating cilia, mucus production, and more robust innate immune responses than conventionally-cultured cells [39]. In ALI culture infected with RV, phosphorylation of LATS1 and STAT1 peaked at day one post infection. LATS1 phosphorylation was no longer observed at days 3 and 5 post-infection, but STAT1 phosphorylation persisted although it was less pronounced than at day 1. For IAV infection, phosphorylation of both LATS1 and STAT1 were observed at days 1, 3, and 5 post infection, albeit with different kinetics than that seen during RV infection. LATS1 phosphorylation was induced by IAV infection and persisted with little change at days 1, 3, and 5 post-infection. STAT1 phosphorylation was seen at day 1 but continued to increase at days 3 and 5 post-infection. The JAK1 inhibitor ruxolitinib blocked both LATS1 and STAT1 phosphorylation, consistent with LATS1 and STAT1 activation by IFN receptor signaling and consistent with our data with recombinant IFNs showing that IFN-mediated LATS1 phosphorylation is JAK1-dependent (Figs 5, 6A, and 6B). We also observed an increase in total STAT1 protein in virus-infected ALI cultures which was abrogated by pre-incubation with ruxolitinib, consistent with STAT1 being a known ISG [1]. To confirm that the increased total STAT1 signal after RV and IAV infection was not due to residual effects of our protocol (blotting for pSTAT1, followed by stringent stripping and probing for total STAT1), we ran a separate gel and western blot for total STAT1 on the same cell lysates without probing for pSTAT1, which also showed an increase in total STAT1 upon infection that was reversed by ruxolitinib pretreatment (S13 Fig). Together, these data are consistent with robust IFN responses to RV and IAV infection in ALI culture with distinct kinetics resulting in different temporal dynamics of LATS1 and STAT1 phosphorylation.

To understand the differences in the dynamics of LATS1 phosphorylation during RV and IAV infection, we next evaluated differences in viral replication kinetics and IFN production between these two infections. RV infection decreased between days 1 and 5 in this model, with live virus shedding detectable at day 1 but not at day 5. In contrast, IAV infection continued to increase between days 1 and 5 (Fig 6C and 6D). There was a trend toward greater cytotoxicity of IAV infection compared to RV infection in ALI culture based on LDH release (S14A–S14D Fig). We also evaluated IFN production induced by these viruses using a reporter assay for Type I IFN and ELISA for Type III IFN (IFNλ1, 2, and 3) that had accumulated in the basolateral media at day 5 post-infection. While both viruses induced robust secretion of Type III IFN, Type III IFN was significantly higher in IAV infection, and Type I IFN activity was only detected in IAV infection (S14E–S14G Fig). These results suggest that differences in viral replication and the magnitude and types of IFN induced may underlie the observed differences in magnitude and kinetics of LATS1 and STAT1 phosphorylation seen during infection of ALI cultures with these two viruses (Fig 6A and 6B).

### The threshold IFN concentration for activation of LATS1 is higher than the threshold for STAT1 activation and is IFN subtype-dependent

**Fig 7:**
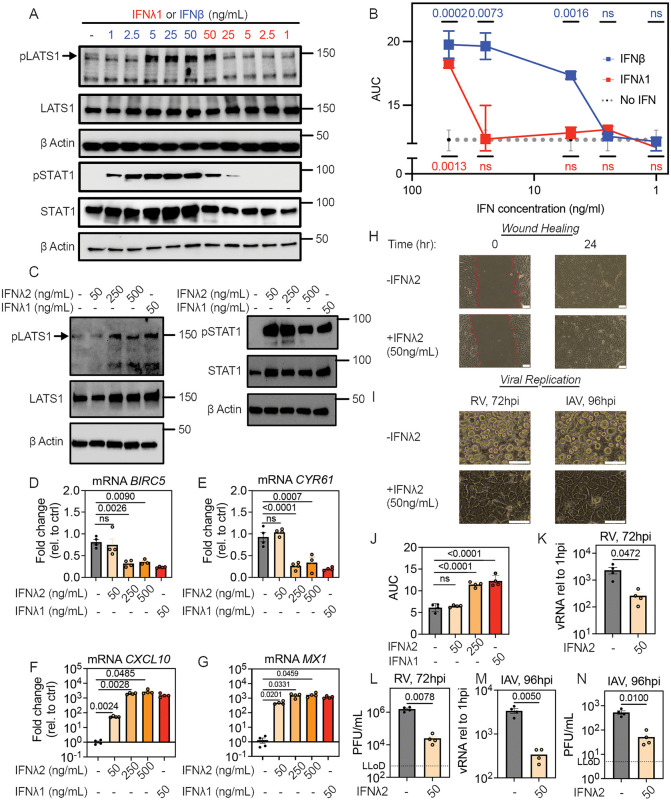
Different threshold doses of IFNs mediate LATS1 and STAT1 phosphorylation. **(A)** Subconfluent HBEC were treated with IFNλ1 (red) or IFNβ (blue) for 6 hours at specified concentrations, followed by western blot on cell lysates for pLATS1 Ser^909^, total LATS1, pSTAT1 Tyr^701^, and total STAT1. Top three panels show western blot for pLATS1/LATS1/β actin and bottom three panels show separate western blot on same cell lysates for pSTAT1/STAT1/β actin. **(B)** Confluent HBEC were treated with IFNλ1 (red) or IFNβ (blue) followed by wounding with a standardized linear scratch (600µm). Fraction of wound area remaining was quantified over time to calculate AUC. Graph shows mean and SEM of three replicates per condition as a function of IFN concentration. AUCs at each IFN concentration were compared using Ordinary One-Way ANOVA test, with red and blue p-values representing comparison between no IFN and IFNλ1 or IFNβ treatment, respectively. **(C–G)** Subconfluent HBEC were treated with IFNλ1 or IFNλ2 at specified concentrations for **(C)** 6 or **(D–G)** 24 hours, followed by **(C)** western blot on cell lysates for pLATS1 Ser^909^, total LATS1, pSTAT1 Tyr^701^, and total STAT1, or **(D–G)** RNA isolation and RT-qPCR for ISGs and YAP target genes. Graphs show mean and SEM of four replicates per condition, with means compared using **(D, E)** Ordinary One-Way ANOVA or **(F, G)** Brown–Forsythe and Welch ANOVA tests. **(H–N)** Confluent HBEC were treated with IFNλ2 or IFNλ1 at specified concentrations prior to **(H, J)** wounding with a standardized linear scratch (600 µm) or **(I, K–N)** infection with RV or IAV. Photomicrographs show **(H)** remaining wound area at 0 and 24 hours after scratch and **(I)** cytopathic effects of viral infection with or without IFNλ2 pretreatment. For all images, scale bar = 100 µm. **(J)** Fraction of wound area remaining was quantified over time to calculate AUC. **(K–N)** RV and IAV replication by RT-qPCR and plaque assay at 72 hours post infection. Graphs show mean and SEM of four replicates per condition and were compared by **(J)** Ordinary One-Way ANOVA test or **(K–N)** Welch’s *t* test. The data underlying this figure can be found in S1 Data and S1 Raw Images.

During physiological viral infections (Fig 6), LATS1 phosphorylation was more restricted in magnitude and duration than STAT1 phosphorylation, prompting us to compare dose–response curves for activation of each signaling event by IFNs. We treated conventionally cultured HBEC with increasing doses of recombinant IFNλ1 or IFNβ ranging from 1 to 50 ng/mL and performed western blot for phosphorylation of LATS1 or STAT1. We observed pLATS1 at 5, 10, and 50 ng/mL of IFNβ but only at 50 ng/mL for IFNL1 (Fig 7A, upper three panels). We observed pSTAT1 across a wider concentration range, at 1, 2.5, 5, 10, and 50 ng/mL of IFNβ and at 25 and 50 ng/mL IFNλ1 (Fig 7A, lower three panels). Consistent with different dose thresholds for LATS1 activation and inhibition of wound healing by IFNβ compared to IFNλ1, in the scratch test assay, 5–50 ng/mL of IFNβ inhibited wound healing, whereas 50 ng/mL IFNλ1 was required to inhibit wound healing (Fig 7B). We also performed a dose–response curve comparing distinct subtypes of Type III IFN: IFNλ1 and IFNλ2. For IFNλ2, 50 ng/mL was insufficient to induce LATS1 phosphorylation in contrast to IFNλ1, but LATS1 phosphorylation was observed with higher concentrations of IFNλ2 (250 or 500 ng/mL) (Fig 7C). These results are consistent with the known lower affinity of IFNλ2 for the Type III IFN receptor compared to IFNλ1 [40]. Notably, IFNλ2 did trigger STAT1 phosphorylation at 50 ng/mL, again indicating a lower threshold for STAT1 than LATS1 activation. To further explore biological consequences, we also examined the concentration threshold for effects of IFNλ1 and IFNλ2 on expression of YAP target genes or ISGs, and on inhibition of wound healing and protection from viral infection. Matching the dose–response curve for LATS1 activation, IFNλ1 inhibited YAP target gene expression at 50 ng/mL, whereas 50 ng/mL of IFNλ2 had no effect (Fig 7D and 7E). In contrast, 50 ng/mL of IFNλ2 did significantly induce ISGs *CXCL10* and *MX1* (Fig 7F and 7G). In the scratch test assay, 50 ng/mL of IFNλ2 did not alter the rate of wound healing, but 50 ng/mL did significantly inhibit viral replication and virus-induced cytopathic effect for both RV and IAV in HBEC (Fig 7H–7N). Together, these data show that IFN-mediated effects on wound healing are both concentration-dependent and IFN subtype-dependent and support the idea that the threshold level of IFN receptor signaling required for IFN-mediated suppression of tissue repair is higher than that required for ISG-mediated antiviral defense.

Together, our results support a model in which high concentrations of IFNs produced during acute infection serve to curtail tissue repair via LATS1 activation until the infection is controlled, while the STAT1-mediated antiviral effects also occur at lower IFN concentrations and continue during the resolution of infection. In addition, activation of LATS1 has also been proposed to promote antiviral defense in some contexts [13,41,42]. To evaluate the antiviral role of LATS1/2 in HBEC, we tested the effect of *LATS1/2* KD on viral load during acute RV or IAV infection. *LATS1/2* KD significantly increased viral replication, leading to a >10-fold increase in viral RNA and a 5- to 10-fold increase in viral titer for both RV and IAV by 72 hours post-infection (S15 Fig). These results suggest that activation of LATS1/2 by high concentrations of IFNs may play role in both regulating the timing of tissue repair and enhancing antiviral defense during acute viral infection.

## Discussion

**Fig 8:**
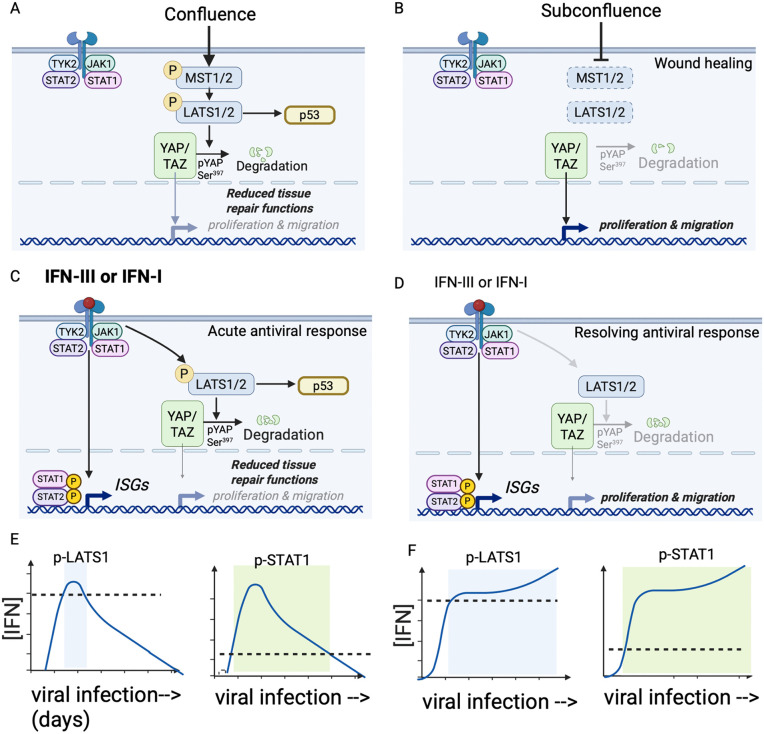
Model. Type III and Type I IFN receptor signaling activate LATS1 to curtail tissue repair during acute viral infection. **(A)** Conditions that signal cell confluence lead to activation and phosphorylation of MST1/2. Active MST1/2 phosphorylate and activate LATS1/2, which then phosphorylate and inactivate YAP, reducing cell proliferation and migration. **(B)** Conditions that signal subconfluence to cells, such as a wound caused by an injury, results in increased YAP nuclear activity, promoting cell proliferation and migration. **(C)** During acute viral infection, high concentrations of Type III and/or Type I IFN receptor signaling trigger JAK-dependent, STAT1-independent phosphorylation of LATS1, leading to phosphorylation and degradation of YAP and reduced tissue repair activities mediated by YAP target genes. In rapidly proliferating cell types, active LATS1/2 also blocks repair in part via a p53-dependent mechanism. **(D)** During resolution of viral infection, lower concentrations of Type III and/or Type I IFN may continue to activate STAT1-mediated antiviral defenses but no longer activate LATS1/2, allowing epithelial repair to resume. **(E–F)** Time course of expected LATS-1 and STAT-1 phosphorylation for viral infections with different kinetics. Dotted lines indicate threshold IFN concentration required for LATS1 activation (high threshold) or STAT1 activation (lower threshold). **(E)** LATS1-P and STAT1-P time course expected for acute viral infection in which viral load and IFN induction peak at day 1 and then decline. **(F)** LATS1-P and STAT1-P time course expected for viral infection that continues to increase in viral load and IFN induction between days 1 and 5 post-infection. *Made with Biorender.com.*

The IFN response is a potent antiviral defense that is rapidly activated when infected cells sense a virus, but its continued activation following acute infection is linked to impaired recovery, as shown in human subjects with severe COVID-19 or influenza infection and in animal models [2–5]. Here, we show that Type I and III IFNs, the two IFN types produced by respiratory epithelial cells in response to viral infection, limit the tissue repair functions of epithelial cells by co-opting the downstream portion of the Hippo signaling pathway, as shown in Fig 8. During development, when cells are confluent, environmental cues lead to activation and phosphorylation of MST1/2 (mammalian homolog of Hippo), which then phosphorylate and activate LATS1/2. Active LATS1/2 then phosphorylate and deactivate YAP, reducing proliferation and migration [12] (Fig 8A). When cells are subconfluent or undergoing repair, this process reverses and YAP nuclear activity increases, driving proliferation and migration (Fig 8B). However, our data indicate that even in subconfluent/repairing cultures, signaling from high concentrations of Type III or Type I IFNs bypass MST1/2 to trigger phosphorylation and activation of LATS1/2, reducing YAP activity, cell migration, and cell proliferation. Our data also indicate that p53, a cell cycle regulator known to be activated by LATS1/2, is also involved in IFN-mediated suppression of epithelial proliferation. We also show that this mechanism is JAK-dependent and STAT1-independent, demonstrating a bifurcation in the signaling pathway: STAT1-independent activation of LATS1 curtails epithelial repair and STAT1-dependent induction of ISGs promotes antiviral defense (Fig 8C). We also show that IFN-mediated LATS1 activation requires a higher threshold concentration of IFN than the pathway leading to STAT1-dependent ISG induction, consistent with the model that inhibition of tissue repair by IFNs only occurs during peak IFN production, whereas the STAT1-mediated ISG response continues to promote antiviral defense even at lower IFN concentrations as seen during resolving infections (Fig 8D). This model is consistent with the distinct kinetics of JAK-STAT1 and JAK-LATS1 activation following an acute viral infection which induces IFNs at the start of infection but then rapidly resolves (Fig 8E), similar to what we observed for rhinovirus infection (Fig 6A and 6C), compared to a viral infection which shows a more sustained increase in viral load and IFN production (Fig 8F), similar to what we observed during influenza infection (Fig 6B and 6D).

Our finding that inhibition of epithelial proliferation and repair is partially p53-dependent in rapidly proliferating bronchial cells fits with a prior study showing that Type I and III IFNs limit lung repair following influenza infection by activating p53 [5]. LATS1/2 activity is known to promote p53-dependent cell cycle arrest [5,43,44]. Our work thus supports a unifying model in which IFN-mediated activation of LATS1 is a common upstream mechanism by which IFN receptor signaling blocks multiple functions associated with tissue regeneration and repair via LATS1, including p53-dependent proliferation and YAP-dependent proliferation and migration.

A strength of this study was the use of primary human cells, and an important contribution of this work is demonstrating that Type III IFN, a specialized IFN type with receptor expression restricted to barrier epithelial cells and a few other cell types, induces phosphorylation of LATS1. Previously, Type I IFN receptor signaling was shown to activate LATS1 in a fibrosarcoma cell line; however, Type III IFN did not have this effect in those experiments despite the cells expressing a functional Type III IFN receptor [13]. Here we show that both IFN types can mediate JAK-dependent activation of LATS1 and STAT1 in primary epithelial cells and that these effects are dose-dependent, suggesting that differences in qualitative effects of IFNs in different cell types could result from quantitative differences in IFN receptor expression levels (among other mechanisms). Our finding that both Type I and Type III IFNs cause LATS1 phosphorylation explains the similar effects of both IFN subtypes on curtailing lung epithelial repair [5]. Furthermore, we observed IFN-dependent, STAT1-independent activation of the Hippo pathway in both airway epithelial cells and primary human fibroblasts, showing that this mechanism is present in more than one cell type that participates in tissue repair and compelling further study of how universal these mechanisms are in other barrier tissues with dynamic repair, such as the skin, gut, and reproductive tract.

These results raise the question of the biological significance of IFN-mediated inhibition of cell proliferation and wound healing. We propose that activation of LATS1 by high concentrations of IFNs produced early in acute infection, but not lower concentrations found during resolving infection, ensures a properly timed progression from antiviral defense to tissue repair during the resolution of acute viral infection. Timing is critical: if repair begins before the infection is fully cleared, the newly regenerated tissue is vulnerable to destruction from the ongoing infection. Type I and Type III IFNs are induced during acute infection but are quickly negatively regulated and therefore well-positioned to serve as a signal that the active infection has not yet resolved. In addition, our data showing that *LATS1/2* KD increases replication of RV or influenza viruses in HBEC suggest that LATS1 activation during peak IFN production also serves another function: promoting heightened antiviral defense. While we did not investigate the mechanism for enhanced viral replication here, prior work shows that LATS1 can reinforce ISG induction by further promoting STAT1 phosphorylation [13]; conversely, YAP activity has been shown to block signaling from viral nucleic acid sensors that initiate the IFN response [15,16], which together with our results indicate reciprocal inhibition between the IFN response and tissue repair responses of epithelial cells.

We speculate that reciprocal inhibition between the IFN response and YAP/TAZ-mediated tissue repair helps to clearly demarcate a switch from prioritizing antiviral defense to prioritizing tissue repair during resolution of an acute viral infection, and that post-viral syndromes associated with sustained IFN production and chronic tissue damage represent a failure to undergo this switch. Intriguingly, a transient increase in antibodies against Type I IFN in the respiratory tract is associated with recovery from mild to moderate COVID-19 infection, suggesting the possibility that transient expression of IFN-neutralizing antibodies could be a mechanism to jumpstart the transition from antiviral defense to tissue repair [45]. Conversely, excessive IFN production has been linked to chronic tissue injury and a failure of wound healing [46,47]. Our model suggests that in conditions of IFN hyperproduction, cells have persistent activation of the downstream portion of the Hippo pathway and thus are unable to properly transition to tissue repair.

An interesting implication of this work stems from the demonstration that the dosage needed to activate the JAK-LATS1 axis differs between IFNβ and IFNλ1, and between IFNλ1 and IFNλ2. An open question in IFN biology is why so many seemingly redundant IFNs are produced during viral infection. Our results suggest that understanding differential activation of LATS1 by different IFNs in specific cell types, for example, due to receptor binding affinity and cell-type-specific receptor expression, may provide new insights into successful versus failed tissue repair post-infection. It will also be important to investigate the molecular reason(s) why JAK1 activates STAT1 at a lower threshold than LATS1, as well as whether similar observations are true across other IFN subtypes.

Here, we focused on the IFNs made by epithelial cells (Type I and Type III), but it will also be important to explore whether Type II IFN (IFNγ) also mediates similar interactions with LATS1. IFNγ is the primary IFN made by T cells responding to acute viral infection and many structural cells, including barrier epithelial cells, express the IFNγ receptor. It will also be interesting to investigate how viral antagonism of IFN receptor signaling influences the JAK-STAT1 and JAK-LATS1 axes in infected versus bystander cells [48,49]. In addition, YAP activity has been shown to participate in cellular differentiation of lung epithelial cells (e.g., goblet cell differentiation and AT2 to AT1 transition) [50,51], which warrants future investigation into the role of IFN-LATS-YAP axis in shaping lung differentiation and remodeling. Together, these studies could help to elucidate the mechanisms by which excessive IFNs hinder proper tissue repair, leading to better targeted therapies to prevent chronic tissue injury post-acute infection or in individuals with IFN-associated autoimmune diseases [52–54].

In sum, this paper reveals LATS1 activation downstream of both Type III and Type I IFN receptor signaling and identifies this interaction as a common regulatory mechanism by which IFNs limit distinct tissue repair functions of respiratory epithelial cells. These results provide a mechanistic model of how IFN signaling regulates both the antiviral response and tissue repair in the airway epithelium and provide a starting point for understanding IFN-associated post-viral tissue damage when the IFN response is disproportionately large or prolonged.

## Materials and methods

### Ethics statement

This project used de-identified primary human bronchial cells obtained from Lonza Bioscience (Walkersville, Maryland, USA) and the BRINDL biobank (University of Rochester, Rochester, NY). Both sources guarantee that all tissue utilized for human cell products are ethically obtained with informed consent in accordance with processes approved by an Institutional Review Board or comparable independent review body. This project also used de-identified primary human wild-type and STAT1 knockout fibroblasts which were ethically obtained with donor-informed consent in accordance with processes approved by an Institutional Review Board as previously described [38].

### Cells and cell culture

Primary HBEC from healthy adults were purchased from a commercial vendor (Lonza, Cat# CC-2450S, Lot numbers 290876; 076043; 023428, 012118; 214917; and 052522). Cells used for S13 Fig were obtained from BRINDL Biobank (donors D180 and D156). Cells were plated on Type I collagen-coated tissue culture polystyrene plates for all experiments except time-lapse microscopy, which used glass 10 mm dishes for the Vivaview system (Mattek, Part# P35GCOL-1.5-10-C). BEGM growth media and supplements were used according to manufacturer’s instructions (Lonza, Cat# CC-3171 and Cat# CC-4175). Cells were used at passage 3 or lower.

For ALI experiments, bronchial epithelial cells were expanded in PneumaCult-Ex Plus Basal Media (STEMCELL Technologies, Cat# 05041). Cells were subsequently differentiated and maintained in PneumaCult-ALI Basal Media (STEMCELL Technologies, Cat# 05002) per manufacturer’s protocol. 24-well, 0.4 µm transparent transwells were obtained from Greiner Bio-One (Lot# 25130116) and coated with collagen prior to use. Bronchial epithelial cells from the BRINDL Biobank were isolated as described previously [55]. Briefly, basal cells were isolated from aliquots of mixed lung cells using anti-Biotin microbeads (Miltenyi Biotec, Cat# 130-090-485), amplified by culturing in BEGM growth media for one passage, then differentiated at ALI.

BCi-NS1.1 cells were a generous gift from Ron Crystal (Weill Cornell Medical College) [32]. Cells were cultured on Type I collagen-coated tissue culture plates using supplemented PneumaCult-Ex Plus Medium per manufacturer’s instructions (STEMCELL Technologies, Cat# 05040).

WT and STAT1^−/−^ human foreskin fibroblasts (HFFs) were a generous gift from Jean-Laurent Casanova (The Rockefeller University) as previously described [38]. HFFs were cultured on Type I collagen-coated tissue culture plates using DMEM (Gibco, Cat# 11965092) supplemented with 10% heat-inactivated fetal bovine serum and 1% penicillin/streptomycin (P/S. HeLa H1 cells were grown in MEM (Gibco Cat# 11095-080) supplemented with 10% heat-inactivated FBS, 1% P/S, and 1% non-essential amino acids.

MDCK cells were grown in DMEM supplemented with 10% FBS and 1% P/S.

### Recombinant interferons and inhibitors

Recombinant IFNs included IFNλ1 (R&D Systems, Cat# 1598-IL-025/CF), IFNλ2 (R&D Systems, Cat# 1587-IL-025), and IFNβ (PBL Assay Science Cat# 11415). For all HBEC and HFF experiments (unless otherwise specified), IFNλ1 and IFNλ2 were diluted in media to a final concentration of 50 ng/mL, and IFNβ was diluted in media to a final concentration of 5ng/mL. For all BCi-NS1.1 experiments, IFNλ1 and IFNβ were diluted in media to final concentrations of 100 ng/mL and 10ng/mL, respectively.

For all conventional culture experiments, ruxolitinib (Cayman Chemicals #11609) and fludarabine (R&D Systems #3495) were diluted in media to 0.5 µM and 1 µM, respectively. STS (Biotium, Cat# 00025) and XMU-MP-1 (Selleck Chem #S8334) were diluted in media to final concentrations of 1 and 10 µM (unless otherwise noted). First, HBEC were pretreated with ruxolitinib for 2 hours, fludarabine for 18 hours, or XMU-MP-1 for 6 hours. Then, media was removed, and new media containing both the inhibitor and IFN was added to each well. To limit the effect of normal cellular processes and proliferation on Hippo pathway activity in WB and RT-qPCR experiments, all wells started with equivalent volumes of media, and IFNs and/or JAK/STAT inhibitors were added at appropriate pretreatment time points. All wells were then collected at the same time. For ALI experiments, cells were pretreated with 50µM ruxolitinib for 18 hours prior to infection.

### Scratch assay

Sixteen hours prior to introducing a scratch (unless otherwise noted in the figure legend), subconfluent cells were treated with recombinant IFN. At the time of the scratch, cells were scratched with a 20 µL pipette tip (Thomas Scientific SHARP, Cat# 1159M43) using a 3D printed apparatus, washed once with warm PBS, then media (+/− IFN) was replaced. Wound healing was observed using inverted microscope (Olympus CKX53 light microscope) or with Vivaview. The VivaView was operated at 5% CO_2_ and a temperature of 37 °C. VivaView images were acquired using a 20×/0.75 DIC Olympus U PlanS APO objective with a WD of 0.65 mm, and a 0.5× magnification lens was used to produce a 10× magnification per field of view. Remaining wound area at specific time points was quantified using MRI Wound Healing Tool Macro (ImageJ/Fiji plugin, RRID: SCR_025260). Key results were repeated in at least three independent experiments using HBEC from at least two different donors.

### Proliferation assays

Subconfluent HBEC were treated with or without IFNλ1 (50 ng/mL) and collected at day zero (prior to the start of IFNλ1 treatment) and days one and two after treatment. Number of living cells was counted using trypan Blue Stain 0.4% (Gibco, Cat# 15250061).

Subconfluent BCi were replaced with or without IFN and collected at day zero (prior to the start of IFN treatment) and day one. At the time of collection, CellTiter-Blue Reagent (Promega, Cat# G8080) was added in 1:5 dilution and incubated in the dark in normal cell culture conditions for two hours, followed by reading by fluorescence (Ex: 540 nm, Em: 590 nm). A hyperbolic line of best fit generated from serial cell dilutions was used to interpolate cell number from fluorescence output. Fold change in cell number was expressed as a proportion of the average initial cell number.

### Western blotting

Cells were harvested using lysis buffer containing Pierce RIPA Buffer (10 mL, Thermo Scientific, Cat# 89900), PhosSTOP (one tablet, Roche, Cat# 04906845001), and cOmplete, Mini Protease Inhibitor Cocktail (one tablet, Roche, Cat# 11697498001).

Protein lysates were loaded onto 4%–20% Mini-PROTEAN TGX-Stain-Free Precast Gels (Bio-Rad Cat# 4568096), run using 1× Tris/Glycine/SDS Buffer prepared from 10× stock (Bio-Rad, Cat# 1610732), and then transferred onto 0.2 µm nitrocellulose membranes (Bio-Rad, Cat# 1704159). Membranes were blocked for 10 minu with EveryBlot Blocking Buffer (Bio-Rad, Cat# 12010020) and incubated with primary antibody overnight at 4 °C, followed by incubation with secondary antibody for 1 hour. Membranes were visualized using Clarity Western ECL Substrate (Bio-Rad, Cat# 1705061) and Bio-Rad ChemiDoc MP Imaging System, and analyzed using Bio-Rad ImageLab software.

Primary antibodies included: YAP (D8H1X, Cell Signaling, #14074, 1:1,000), phospho-YAP Ser^397^ (D1E7Y, Cell Signaling, #13619, 1:1,000), phospho-LATS1 Ser^909^ (Cell Signaling, #9157, 1:1,000), LATS1 (C66B5, Cell Signaling, #3477, 1:1,000), LATS2 (D83D6, Cell Signaling, #5888, 1:1,000), phospho-MST1 Thr^183^/MST2 Thr^180^ (Cell Signaling, #49332, 1:1,000), MST1 (Cell Signaling, #14946, 1:1,000), JAK1 (Cell Signaling, #3344, 1:1,000), phospho-STAT1 Tyr^701^ (Cell Signaling, #9167, 1:1,000), STAT1 (Cell Signaling, #9172, 1:1,000), Flag Tag (ThermoFisher, Ref# MA1-91878, 1:2,000), P53 (Cell Signaling, #2527, 1:1,000), and β Actin (Santa Cruz, sc-47778, 1:1,000). The phospho-LATS1 Ser^909^ antibody recognized a band at 150 kDa (the correct molecular weight, denoted with arrows) and additional bands at approximately 50 and 75 kDa. The JAK1 antibody recognized a band at 130 kDa (the correct molecular weight, denoted with arrow) and a non-specific band at approximately 100 kDa.

Secondary antibodies included: Goat anti-Rabbit IgG (Jackson ImmunoResearch, Cat# 111-035-003, 1:5,000–1:2,000) and Goat anti-Mouse IgG (Jackson ImmunoResearch, Cat# 115-035-003, 1:5,000). Both antibodies were prepared following manufacturer instructions.

### Viral infections

Submerged HFF and HBEC were incubated in 24-well plates with influenza A virus (IAV) H1N1pdm09 (ATCC, VR-1894, #70014833) or IAV PR8-GFP, graciously provided by the García-Sastre Laboratory [37], respectively for one hour in 200 µl PBS with 0.1% BSA at MOI of 0.1, 37 °C. HFF and HBEC were similarly infected with rhinovirus strain A1 (ATCC, VR-1559, #57685054) for one hour at MOI of 0.05, 33 °C. After inoculation period, inoculum was removed, and fresh media was added. For HFF infected with IAV, fresh media also contained 0.1% TPCK-trypsin.

For IAV PR8-GFP infection, RNA and supernatant were collected at 1 and 24hpi. For IAV H1N1pdm09 and RV-A1 infections, RNA and supernatant were collected at specified time points. vRNA was analyzed by RT-qPCR. To evaluate the effect of IFNs on viral replication, HBEC were treated with IFNλ1 or IFNλ2 for 18 hours prior to infection; HFF were pretreated with IFNβ for four hours prior to infection. No IFNs were added after infection.

For ALI experiments, IAV H1N1pdm09 and RV-A1 were used at MOI of 0.25, 35 °C. At 3 dpi, an additional 200 µL of basolateral media was added to each well. Apical wash, collected by washing cells once with 200 µL cold PBS, was used for plaque assay. Cell lysate was collected for western blot. Basolateral media was collected for LDH assay (Cayman Chemical #601170, as described in manufacturer’s protocol, with values calculated from standard curve of lysed cells), Type I IFN reporter assay (InvivoGen, Cat# hkb-ifnabv2), and Type III IFN ELISA (PBL Assay Science, Cat# 61840).

For IAV PR8-GFP, we used confocal microscopy (see below) to quantify the number of infectious particles. For RV-A1, we performed plaque assays as previously described [56]. For IAV H1N1pdm09, we performed plaque assays as follows. Briefly, MDCK cells in 6-well plates were inoculated with 200 µL of supernatant (conventional culture) or apical wash (ALI culture) serially diluted in PBS + 0.1% BSA for 1 hour with rocking, then overlaid with plaque assay medium (1× MEM [Gibco Ref#11935-046]), 0.07% BSA/PBS, 1 µg/mL TPCK-treated trypsin, 0.3% agarose) and incubated at 37 °C for three days.

### Confocal microscopy

For YAP localization experiments, cells were seeded on collagen-coated coverslips and grown to confluence with or without treatment of ruxolitinib/fludarabine or *LATS1/2* siRNA. Cells were scratched as described above and fixed four hours after the scratch. Samples were stained as described in Cell Signaling immunofluorescence protocol. Reagents used: normal goat serum (Sigma-Aldrich, G6767), YAP (D8H1X, Cell Signaling, #14074, 1:200), Goat Anti-Rabbit IgG H&L (Abcam, Cat# ab150079, 1:200), and counterstaining with 2.5 mg/mL DAPI (BioLegend, Cat# 422801). Samples were analyzed using 20× objective on Zeiss LSM 880 Airyscan confocal microscope and Zen Black software. ImageJ was used to split the channels and quantify the number of cells with co-localized YAP and DAPI.

For IAV PR8-GFP infection, HBEC grown on collagen-coated coverslips were infected as described above. After 24 hours, cells were washed twice with PBS and fixed with 3.7% formaldehyde for 15 min. Coverslips were then washed three times with PBS, counterstained with 2.5 mg/mL DAPI for 10 min, and washed three more times with PBS (all steps on ice). GFP-positive cells per high power field were counted using 10× objective on Zeiss LSM 880 Airyscan confocal microscope and Zen Black software. Images were processed using ImageJ.

Key results were repeated in at least three independent experiments using HBEC from at least two different donors.

### siRNA knockdowns, RT-qPCR protocol, and primers

siRNAs were purchased from Dharmacon—control (D-001220-01), *IFNLR1* (D-007981-01 and D-007981-02), *LATS1* (J-004632-05-002), *LATS2* (L-003865-00-0005), *JAK1* (D-003145-05-0002), and *TP53* (D-003329-05-0002)—and transfected into cells using DharmaFECT (GE Dharmacon, cat# T-2001-02) per manufacturer’s instructions. The siRNA targeting *LATS1* and *LATS2* were chosen based on previous work showing an effective KD [57]. Cells were 40% confluent at time of transfection. Transfection was performed for 6 hours at 37 °C, then cells were replaced with fresh media. Cells were incubated for three more days with twice-daily media changes prior to start of experimentation. Key results were repeated in at least three independent experiments using HBEC from at least two different donors.

RT-qPCR was performed as previously reported [58]. For all RT-qPCR experiments, mRNA levels of host genes were normalized to housekeeping gene HPRT and are graphed as fold change from mock-treated cells or are presented relative to the level of the target mRNA relative to HPRT. Primer sequences were as follows in Table 1

**Table 1:** Primer sequences.

Target	Primer sequence
HPRT Forward	5′-TGGTCAGGCAGTATAATCCAAAG-3′
HPRT Reverse	5′-TTTCAAATCCAACAAAGTCTGGC-3′
CXCL10 Forward	5′-CCTGCAAGCCAATTTTGTCC-3′
CXCL10 Reverse	5′-ATGGCCTTCGATTCTGGATTC-3′
MX1 Forward	5′-AGAGAAGGTGAGAAGCTGATCC-3′
MX1 Reverse	5′-TTCTTCCAGCTCCTTCTCTCTG-3′
IFNLR1 Forward	5′-CCAGATCACTCTCCAGCCAG-3′
IFNLR1 Reverse	5′-GGTGGGCTTAGAGAACTTGC-3′
CYR61 Forward	5′-GTTACCAATGACAACCCTGAGT-3′
CYR61 Reverse	5′-TGCATTTCTTGCCCTTTTTCAG-3′
BIRC5 Forward	5′-CTGAGAACGAGCCAGACTTG-3′
BIRC5 Reverse	5′-TATGTTCCTCTATGGGGTCGT-3′
TRIO Forward	5′-CTTCTTCCGATCCGGGTTTC-3′
TRIO Reverse	5′-CTCCCACCTGAAAGGTCTGC-3′
CTGF Forward	5′-AGAGCAGCTGCAAGTACCAG-3′
CTGF Reverse	5′-GTAATGGCAGGCACAGGTCT-3′
EGR3 Forward	5′-CAATCTGTACCCCGAGGAGAT-3′
EGR3 Reverse	5′-CCGATGTCCATTACATTCTCTGTA-3′
VIM Forward	5′-CTGCAATCTTTCAGACAGGATGT-3′
VIM Reverse	5′-GTGGAGTTTCTTCAAAAAGGCAA-3′
LATS1 Forward	5′-GCTCTCCCCTCCAGAGTTA-3′
LATS1 Reverse	5′-TCCAGAGCTTTCTTCTGAGC-3′
LATS2 Forward	5′-CGCCCCTGGAGAGAGTG-3′
LATS2 Reverse	5′-CCTTTTGAAAATGTTCTTTCCTTCC-3′
ISG15 Forward	5′-CATCTTTGCCAGTACAGGAGC-3′
ISG15 Reverse	5′-GGGACACCTGGAATTCGTTG-3′
OASL Forward	5′-AAGGTAGTCAAGGTGGGCTC-3′
OASL Reverse	5′-CTCCTGGAAGCTGTGGAAAC-3′
RV-A1 Forward	5′-CAGGCCAAATTAAAGTCAATAAGC-3′
RV-A1 Reverse	5′-AGGCTGAAGTTTGGTTTTGC −3′
IAV PR8-GFP Forward	5′-ATACCCAAGCAGAAAGTGGC-3′
IAV PR8-GFP Reverse	5′-AGCCGGTCAAAAATCACACT-3′
IAV Forward	5′-TGCCGGTTTCATTGAAGGGG-3′
IAV Reverse	5′-CTCTTCAGGTCGGCTGCATA −3′

### BCi construct overexpression protocol

BCi were transduced either with an empty control vector (PGKp-GFP-Empty, Addgene plasmid# 174175) or YAP5SA (PGKp-GFP-YAP (5SA), Addgene plasmid# 174174), as previously described [33]. Successful transduction of both constructs was confirmed by sorting based on GFP expression. Successful transduction of GFP-YAP (5SA) was also validated by western blot against Flag tag.

### Statistical analysis

All statistical analysis was done using GraphPad Prism 10. Statistical tests used are indicated in figure legends and S1 Data file. For wound healing assays, rate of wound healing was summarized as the AUC of the graph of time (*x*) versus fraction of wound area remaining (*y*). The total peak area was used, defined as the AUC of all points at ≥10% of the distance from minimum to maximum y.

## Supporting information

S1 Fig Effect of IFNλ1 on HBEC proliferation over 48 hours. Subconfluent HBEC were treated with IFNλ1 (50ng/mL), and number of cells was counted at 24-hour intervals using a hemocytometer on wheels. Graph shows mean of 3 (*t* = 0) or 6 (*t* = 24, 48 hours) biological replicates per condition. Mock- and IFNλ1-treated conditions were not significantly different by Welch’s *t* test. The data underlying this figure can be found in S1 Data.
(TIF)

S2 Fig Type III IFN receptor knockdown rescues the effect of IFNλ1 on wound healing. **(A–C)** Subconfluent HBEC were transfected with either control or Type III IFN receptor (IFNLR1)-targeting siRNA, allowed to recover for three days, then treated with IFNλ1 for 6 hours, followed by RNA isolation and RT-qPCR for *CXCL10*, *MX1*, and *IFNLR1* to assess knockdown efficiency. Graphs show mean and SEM of four replicates per condition, with means compared using (A,B) Ordinary One-Way ANOVA test or (C) Mann–Whitney test. **(D)** Confluent siCtrl and siIFNLR1 HBEC were treated with IFNλ1 followed by wounding with a standardized linear scratch (600 µm). Fraction of wound area remaining was quantified over time to calculate AUC. Graph shows mean and SEM of four replicates per condition, with means compared using Ordinary One-Way ANOVA test. The data underlying this figure can be found in S1 Data.
(TIF)

S3 Fig IFNβ inhibits YAP nuclear activity in subconfluent HBEC cultures. Subconfluent HBEC were treated with IFNβ for specified times, followed by RNA isolation and RT-qPCR for YAP target genes **(A)** *CYR61* or **(B)** *BIRC5*. Graphs show mean and SEM of six replicates per condition, with means compared using Ordinary One-Way ANOVA. The data underlying this figure can be found in S1 Data.
(TIF)

S4 Fig IFNλ1 and IFNβ decrease expression of YAP-dependent, but not YAP-independent, genes involved with migration. Subconfluent HBEC were treated with IFNλ1 (red) or IFNβ (blue) for 24 hours, followed by RNA isolation and RT-qPCR for **(A, B)** YAP target genes involved with cell migration, and **(C, D)** YAP-independent genes involved with cell migration. Graphs show mean and SEM of six replicates per condition, with means compared using Ordinary One-Way ANOVA test. The data underlying this figure can be found in S1 Data.
(TIF)

S5 Fig IFNλ1 at the time of the scratch is sufficient for suppression of wound healing. **(A)** Schematic of experiment. Cells were treated as follows: mock (black), IFNλ1 treatment 16 hours prior to scratch (*t* = −16 h) and at the time of the scratch (*t* = 0 h) (red), or IFNλ1 treatment at the time of the scratch only (*t* = 0 h) (cyan). **(B)** Confluent HBEC treated as in (A) were wounded with a standardized linear scratch (600 µm). Fraction of wound area over time was quantified to calculate AUC. Graph shows mean and SEM of four replicates per condition, with means compared using Ordinary One-Way ANOVA test. The data underlying this figure can be found in S1 Data.
(TIF)

S6 Fig Validation of LATS1/2 siRNA knockdown. HBEC were transfected with control or LATS1/2 siRNA, followed by **(A, B)** RNA isolation and RT-qPCR for *LATS1* and *LATS2* one day post-transfection. Graphs show mean and SEM of six replicates per condition, with means compared using Brown–Forsythe and Welch ANOVA tests. **(C)** western blot on cell lysates for total LATS1 and LATS2 four days post-transfection. The data underlying this figure can be found in S1 Data and S1 Raw Images.
(TIF)

S7 Fig MST1 is not required for IFN-mediated LATS1 activation, inhibition of YAP target gene expression, or suppression of wound healing. **(A)** Subconfluent HBEC were treated with IFNλ1 (red) or IFNβ (blue) for specified times, followed by western blot on cell lysates for pMST1 Thr^183^ and total MST1. STS: staurosporine. **(B)** Subconfluent HBEC were pretreated with XMU-MP-1 (XMU) at concentrations of 1, 6, 10, and 50 µM prior to STS treatment, followed by western blot on cell lysates for pMST1 Thr^183^ and total MST1. **(C)** Subconfluent HBEC were pretreated with XMU-MP-1 (XMU) prior to treatment with STS for three hours or IFNλ1 for six hours, followed by western blot on cell lysates for pLATS1 Ser^909^ and total LATS1. **(D, E)** Subconfluent HBEC were pretreated with XMU prior to treatment with IFNλ1 or IFNβ for 24 hours, followed by RNA isolation and RT-qPCR for *CYR61* or *BIRC5*. **(F)** Confluent HBEC were pretreated with XMU, followed by IFNλ1 or IFNβ treatment and wounding with a standardized linear scratch (600 µm). Fraction of wound area remaining was quantified over time to calculate AUC. Graphs show mean and SEM of four replicates per condition, with means compared using (D, E) Brown–Forsythe and Welch ANOVA or (F) Ordinary One-Way ANOVA tests. The data underlying this figure can be found in S1 Data and S1 Raw Images.
(TIF)

S8 Fig Both ruxolitinib and fludarabine attenuate ISG induction after IFNλ1 treatment in HBEC. Rux- and flud-pretreated HBEC were treated with IFNλ1 for 24 hours, followed by RNA isolation and RT-qPCR for ISGs. Graphs show mean and SEM of six replicates per condition, with means compared using Brown–Forsythe and Welch ANOVA tests. The data underlying this figure can be found in S1 Data.
(TIF)

S9 Fig Inhibiting JAK1, but not STAT1, rescues effect of IFNλ1 on wound healing by video microscopy. Sham-, rux-, or flud-pretreated HBEC were grown to confluence in 10 mm glass plates, then wounded with a standardized linear scratch (300 µm). Fraction of wound area remaining with and without IFNλ1 pretreatment was calculated using time lapse microscopy. Three replicate wells per condition are shown, with area under the curve (AUC) compared using Welch’s *t* test. The data underlying this figure can be found in S1 Data.
(TIF)

S10 Fig Fludarabine treatment compromises IFNλ1-mediated suppression of viral replication. Sham- or flud-pretreated HBEC were treated with or without IFNλ1 prior to inoculation with rhinovirus (RV) or influenza A-PR8 GFP virus (IAV PR8-GFP) **(A, B)** RV viral load by RT-qPCR and plaque assay at 72hpi. **(C–E)** IAV PR8-GFP viral load by RT-qPCR or GFP+ cells per high power field at 24hpi. **(E)** Micrographs of IAV PR8-GFP infected cultures, 24hpi. Green: IAV PR8-GFP, blue: DAPI. Scale bar = 100 µm. Graphs show mean and SEM of **(A–C)** four or **(D)** eight replicates per condition, with means compared using Brown–Forsythe and Welch ANOVA tests. The data underlying this figure can be found in S1 Data and at https://doi.org/10.17632/6rvkfsy2n8.1.
(TIF)

S11 Fig IFNβ curtails wound healing but not respiratory virus replication in STAT1-deficient primary human fibroblasts. **(A–D)** Subconfluent WT and STAT1^−/−^ cells were treated with IFNλ1 (red) or IFNβ (blue) for 24 hours, followed by RNA isolation and RT-qPCR for ISGs and YAP target genes. Graphs show mean and SEM of four replicates per condition and are compared using **(A)** Brown–Forsythe and Welch ANOVA or **(B–D)** Ordinary One-Way ANOVA tests. **(E, F)** Confluent WT and STAT1^−/−^ cells were pretreated with or without IFNβ and wounded with a standardized linear scratch (600 µm). Graphs show fraction of wound area at times indicated and mean and SEM of four replicates per condition, with AUCs compared using Welch’s *t* test. **(G–I)** WT and STAT1^−/−^ cells were pretreated with IFNβ for four hours prior to infection with **(G, H)** RV1A or **(I)** IAV. Viral RNA and titer were quantified at 72hpi. Graphs show mean and SEM of four replicates per condition, with means compared using Brown–Forsythe and Welch ANOVA tests. The data underlying this figure can be found in S1 Data.
(TIF)

S12 Fig JAK1 knockdown in primary HBEC leads to loss of JAK1 protein and abrogates IFNλ1-mediated ISG induction. **(A)** Subconfluent HBEC were transfected with siCtrl or siJAK1, followed by western blot on cell lysates for JAK1. **(B, C)** Subconfluent HBEC were treated with IFNλ1 **(B–E)** for 24 hours, followed by RNA isolation and RT-qPCR for ISGs. Graphs show mean and SEM of four replicates per condition, with means compared using **(A)** Brown–Forsythe and Welch ANOVA or **(B)** Ordinary One-Way ANOVA tests. The data underlying this figure can be found in S1 Data and S1 Raw Images.
(TIF)

S13 Fig Total STAT1 protein increases after RV or IAV infection and is abrogated by ruxolitinib. Air–liquid interface HBEC cultures were mock-pretreated or pretreated with ruxolitinib prior to infection with RV or IAV. Western blots of cell lysates show total STAT1 and β actin. These membranes were not probed for pSTAT1 Tyr^701^. The data underlying this figure can be found in S1 Raw Images.
(TIF)

S14 Fig Viral titer, cytopathic effects, and IFN induction following RV and IAV infection in ALI culture. **(A–D)** RV- and IAV-infected **(A, B)** apical wash were collected for plaque assay and **(C, D)** basolateral media were collected for LDH assay at 1 and 5 dpi. **(E)** Standard curve of Type I IFN reporter cells, with absorbance at 630nm corresponding to Type I IFN activity, showing mean and SEM of three replicates per standard. **(F, G)** RV- and IAV-infected basolateral media were collected for **(F)** Type I IFN reporter assay (HEK-Blue reporter from Invivogen) and **(G)** Type III IFN ELISA (for IFNλ1, 2, and 3) at 1 and 5 dpi. **(A–D, F, G)** Graphs show mean and SEM overlayed with three replicates and were compared using **(A–D)** Ordinary One-Way ANOVA test or **(F, G)** unpaired *t* test. The data underlying this figure can be found in S1 Data.
(TIF)

S15 Fig LATS1/2 knockdown promotes rhinovirus and influenza A virus replication. siCtrl and siLATS1/2-transfected HBEC were infected with **(A, B)** rhinovirus or **(C, D)** influenza A virus for 72hpi, followed by RNA isolation and RT-qPCR for vRNA, and plaque assay for viral titer. Graphs show mean and SEM of four replicates per condition, with means compared using Welch’s *t* test. The data underlying this figure can be found in S1 Data.
(TIF)

S1 Data Individual values underlying the graphs shown in the figures along with the corresponding statistical analysis. (XLSX)

S1 Raw Images Uncropped blots used to create Figs 2–7, S6, S7, S12, and S13. (PDF)

S1 Video Time-lapse video of wound healing of HBEC over 300µm scratch (−IFNλ1). Images were taken every 15 min over 48 hours, as indicated in time stamp (upper right). Resolution is 0.549 µm/pixel. Scale bar = 100μm.
(MP4)

S2 Video Time-lapse video of wound healing of HBEC over 300 µm scratch (+IFNλ1). Images were taken every 15 min over 48 hours, as indicated in time stamp (upper right). Resolution = 0.549 µm/pixel. Scale bar = 100 μm.
(MP4)

## Abbreviations

ALI: air–liquid interface
AUC: area under the curve
HBEC: human bronchial epithelial cells
HFFs: human foreskin fibroblasts
IFNs: interferons
ISGs: , interferon-stimulated genes
JAKs: Janus kinases
KD: knockdown
STS: staurosporine
TAZ: transcriptional co-activator with PDZ-binding motif
YAP: Yes-associated protein
