# Supplementary material for: Dose-dependent activation of the Hippo pathway by Type I and Type III interferons suppresses tissue repair by human bronchial epithelial cells
Source: PLoS Biol. 2026 Jan 26;24(1):e3003615. doi: 10.1371/journal.pbio.3003615 (PMC12863674; doi:10.1371/journal.pbio.3003615)

Figure 2A

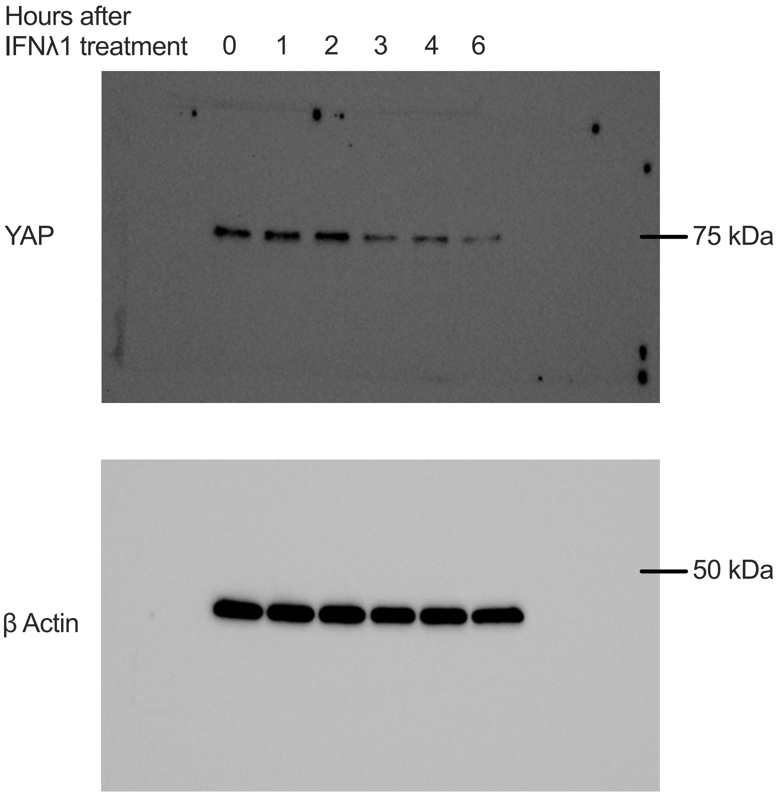

Figure 2B

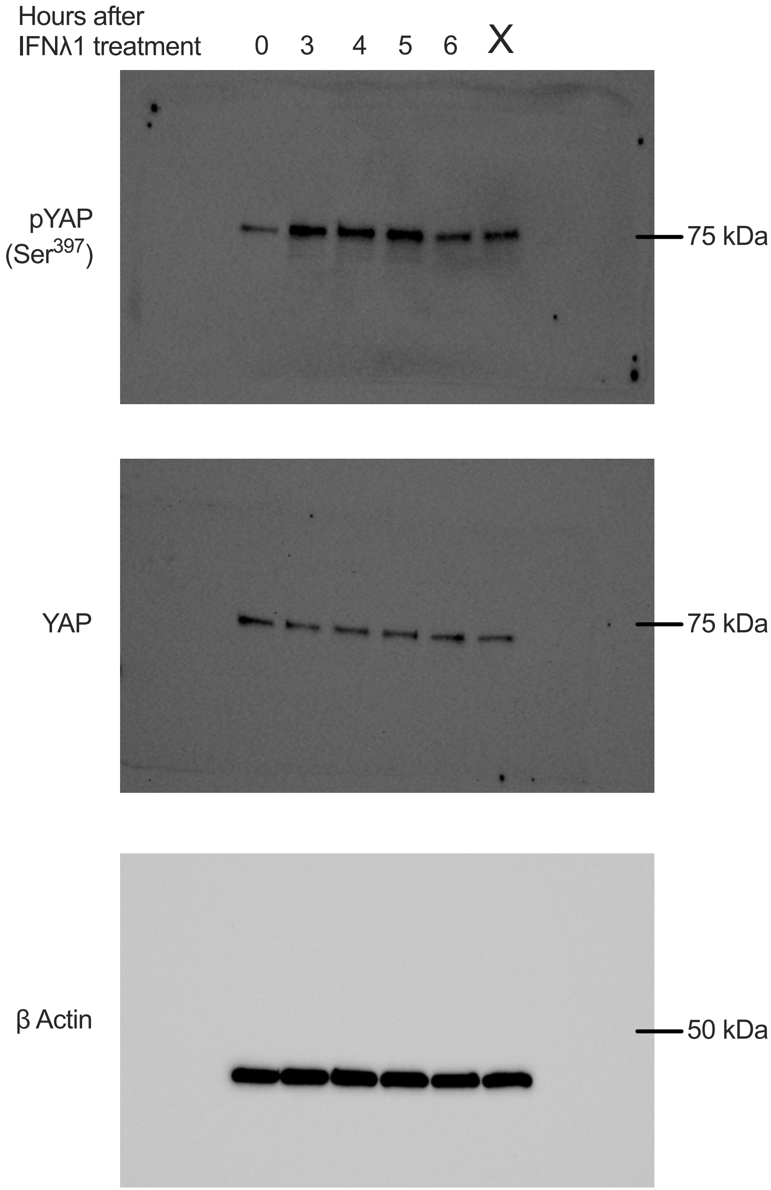

Figure 3A

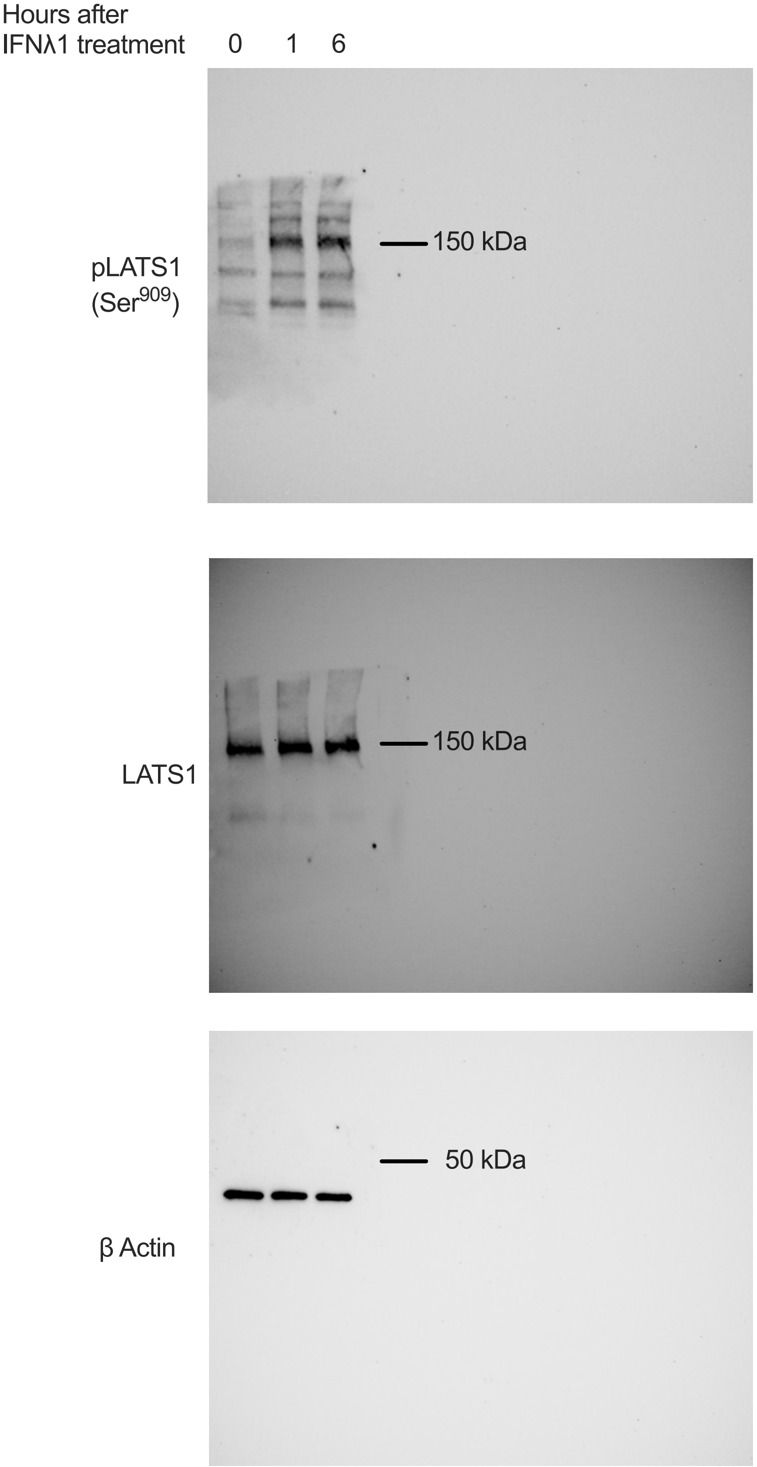

Figure 3B

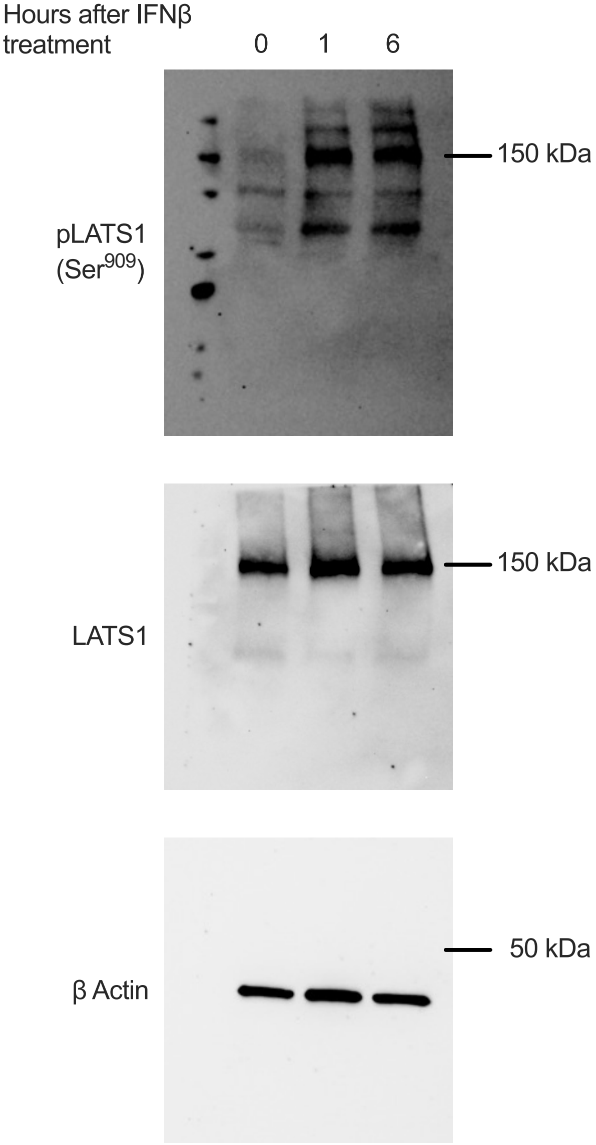

Figure 4E

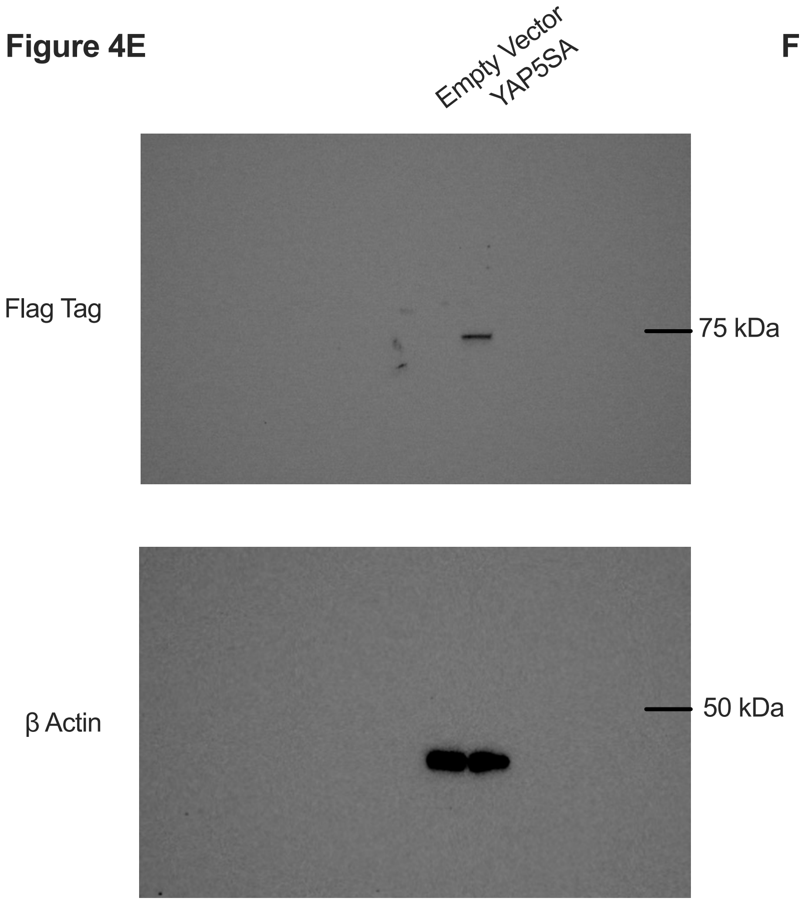

Figure 4H

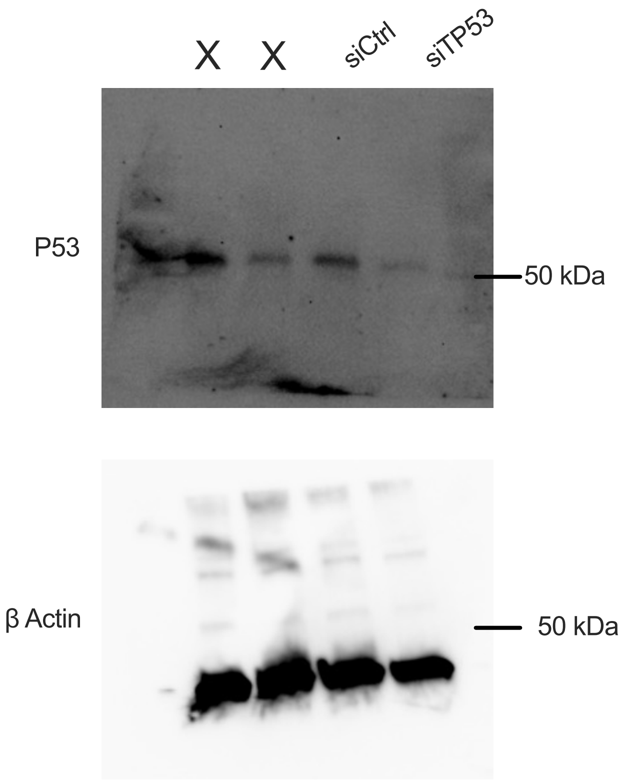

Figure 5B

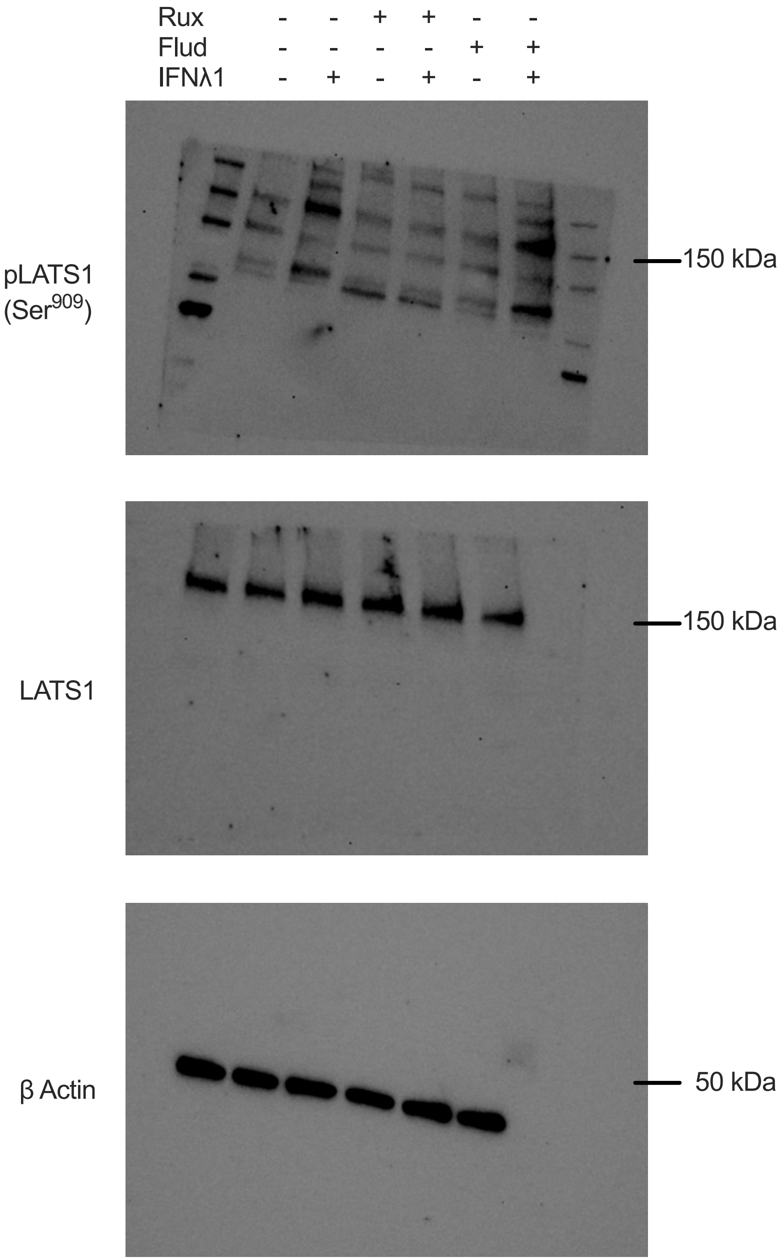

Figure 5O

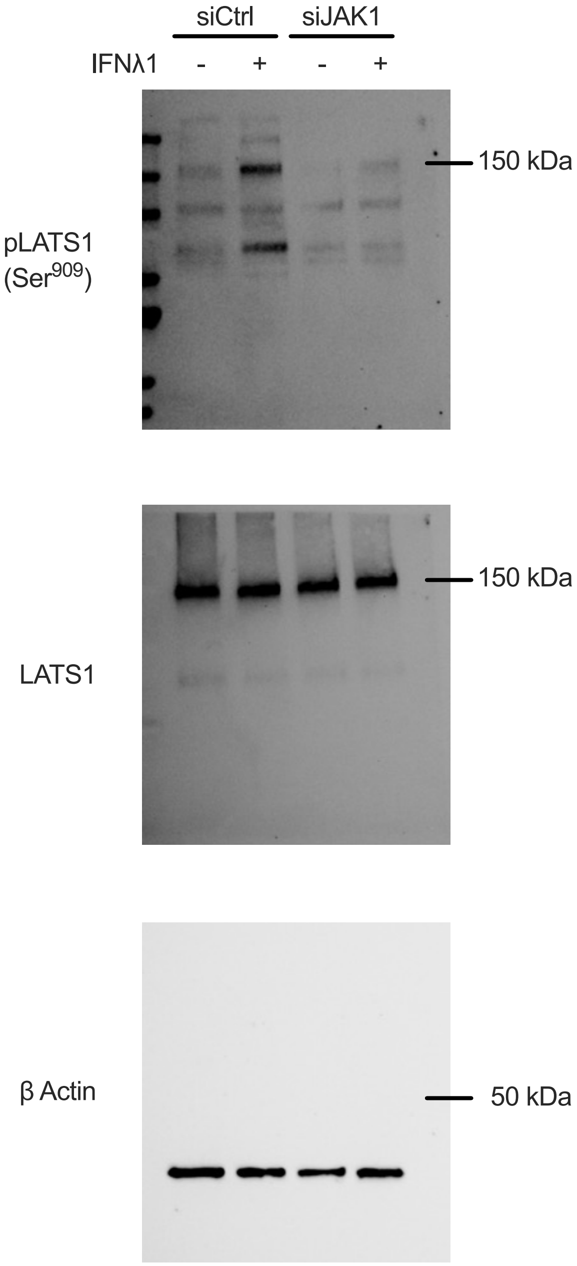

Figure 6A

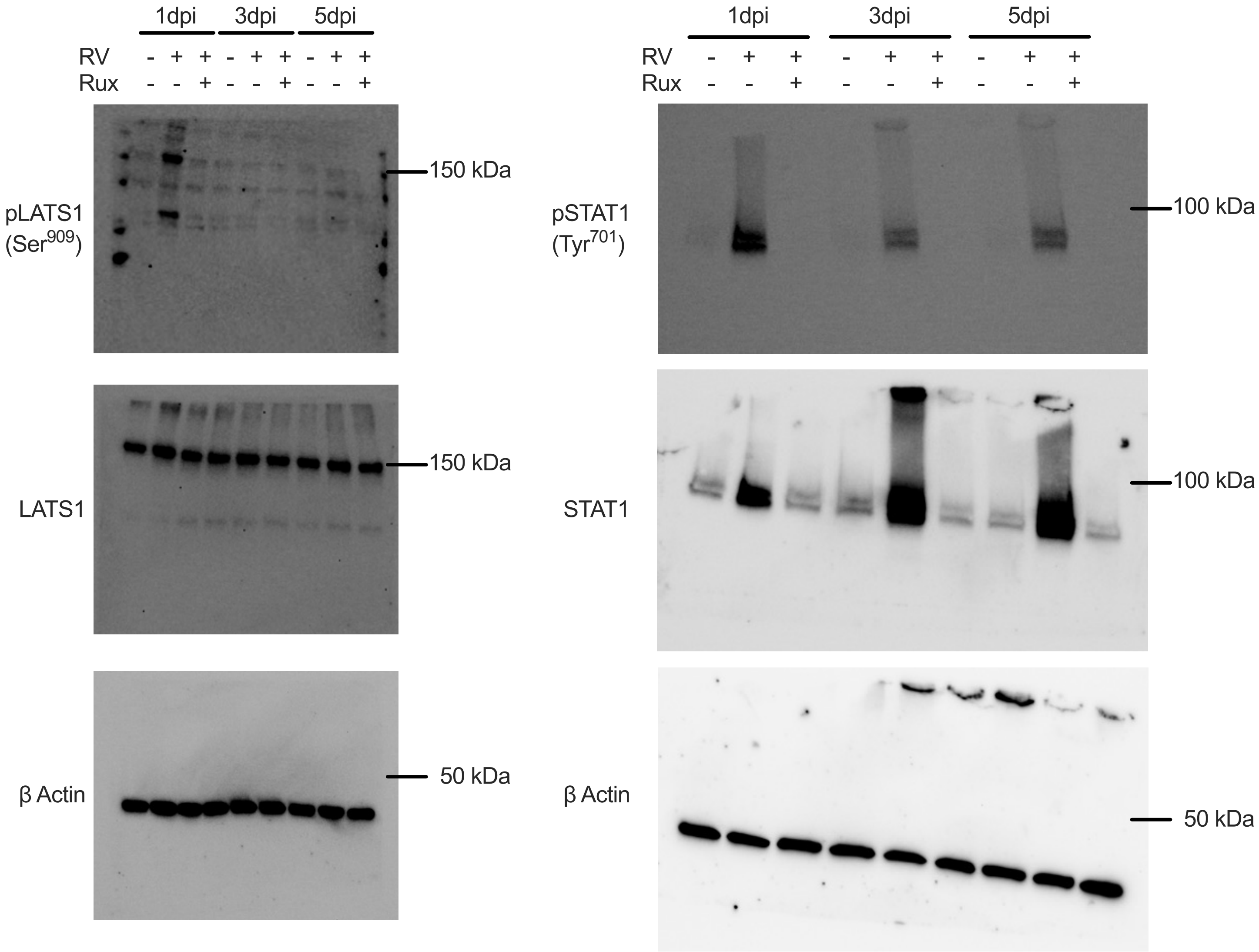
1dpi
3dpi
5dpi
RV - + + - + + - + +
Rux - - + - - + - - +
pLATS1 (Ser909)
150 kDa
LATS1
150 kDa
β Actin
50 kDa
1dpi
3dpi
5dpi
RV - + + - + + - + +
Rux - - + - - + - - +
pSTAT1 (Tyr701)
100 kDa
STAT1
100 kDa
β Actin
50 kDa

Figure 6B

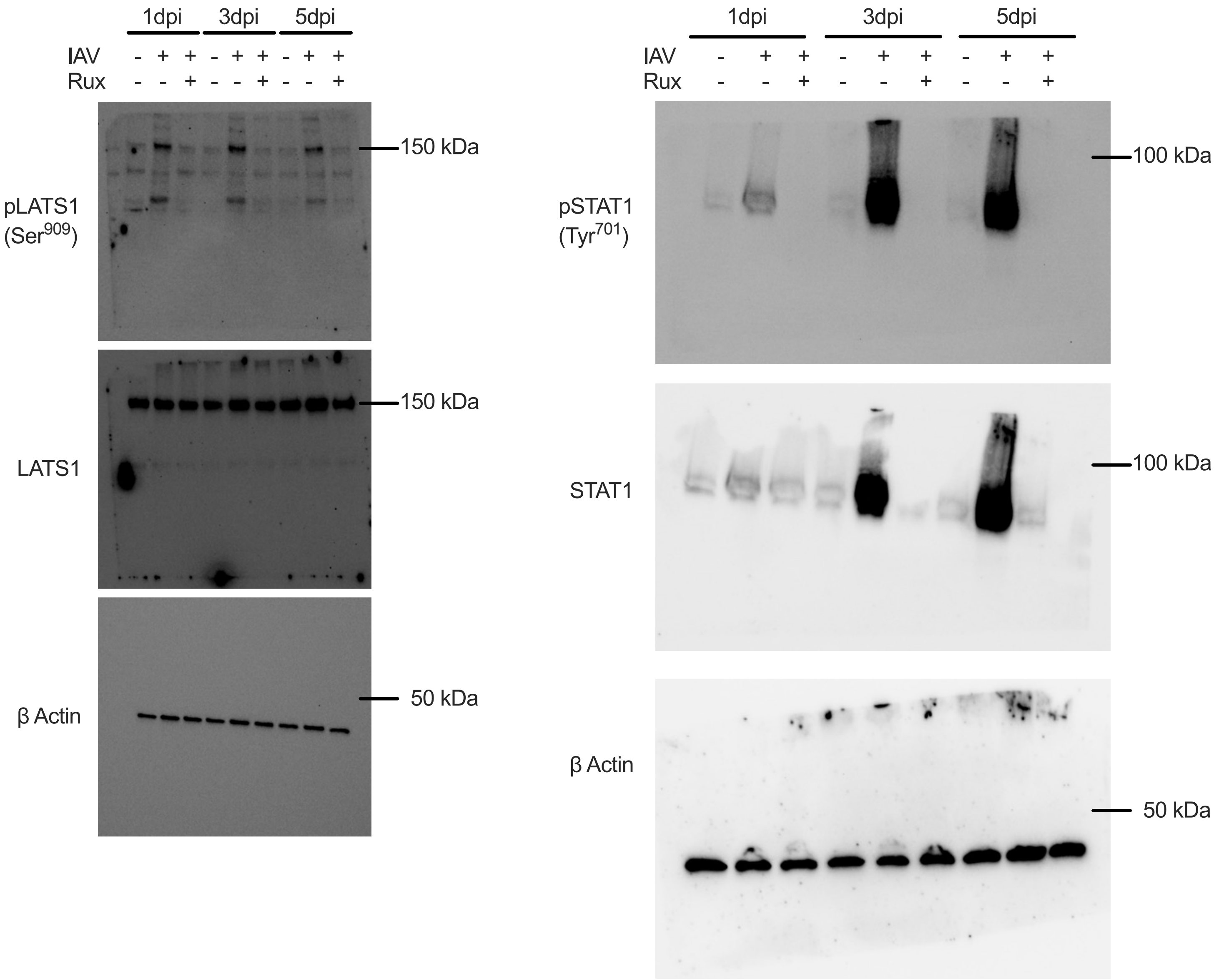
1dpi
3dpi
5dpi
IAV - + + - + + - + +
Rux - - + - - + - - +
pLATS1 (Ser909)
150 kDa
LATS1
150 kDa
β Actin
50 kDa
1dpi
3dpi
5dpi
IAV - + + - + + - + +
Rux - - + - - + - - +
pSTAT1 (Tyr701)
100 kDa
STAT1
100 kDa
β Actin
50 kDa

Figure 7A

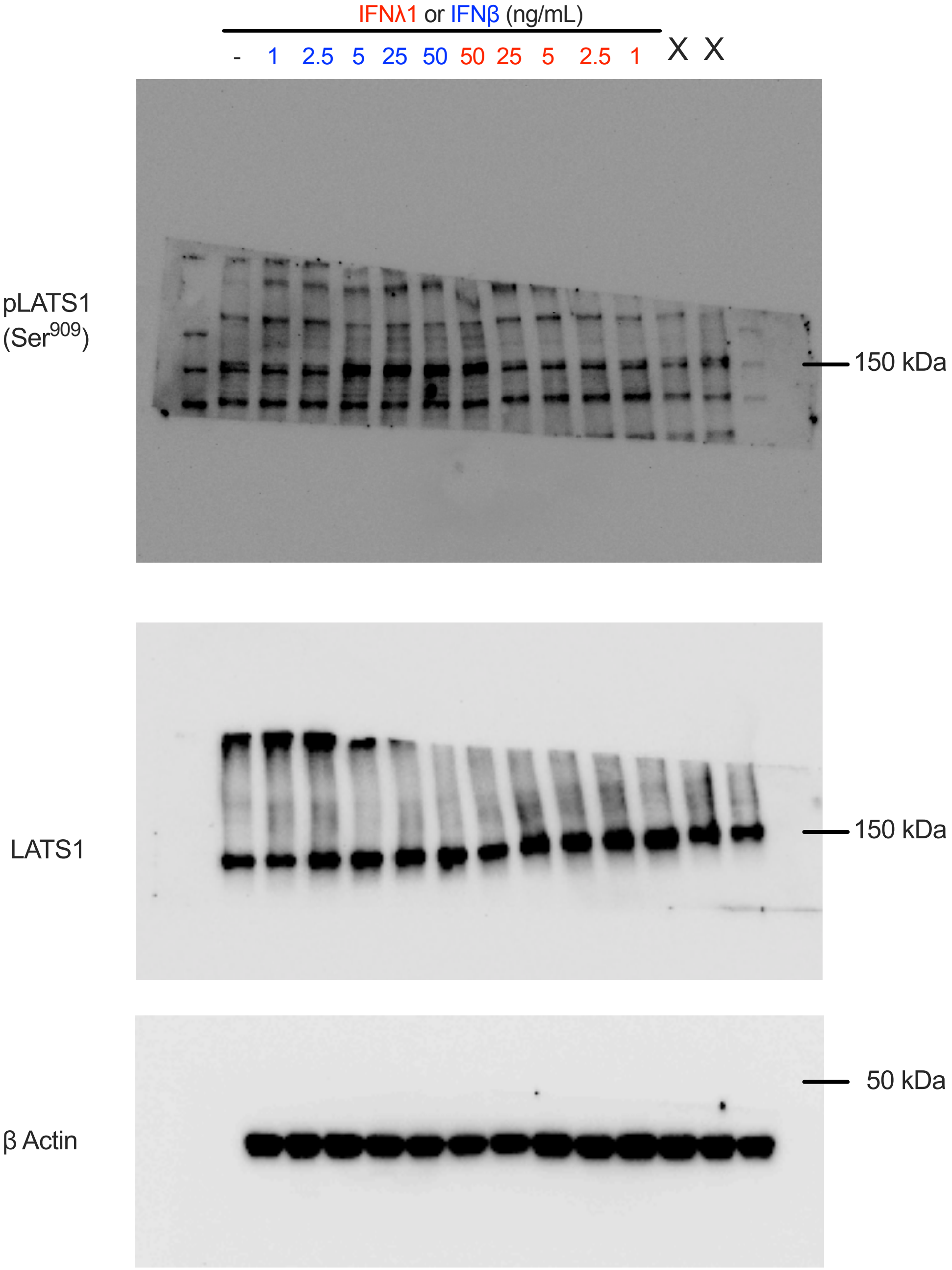
IFNλ1 or IFNβ (ng/mL)
- 1 2.5 5 25 50 50 25 5 2.5 1 X X
pLATS1 (Ser909)
150 kDa
LATS1
150 kDa
β Actin
50 kDa

Figure 7A part 2

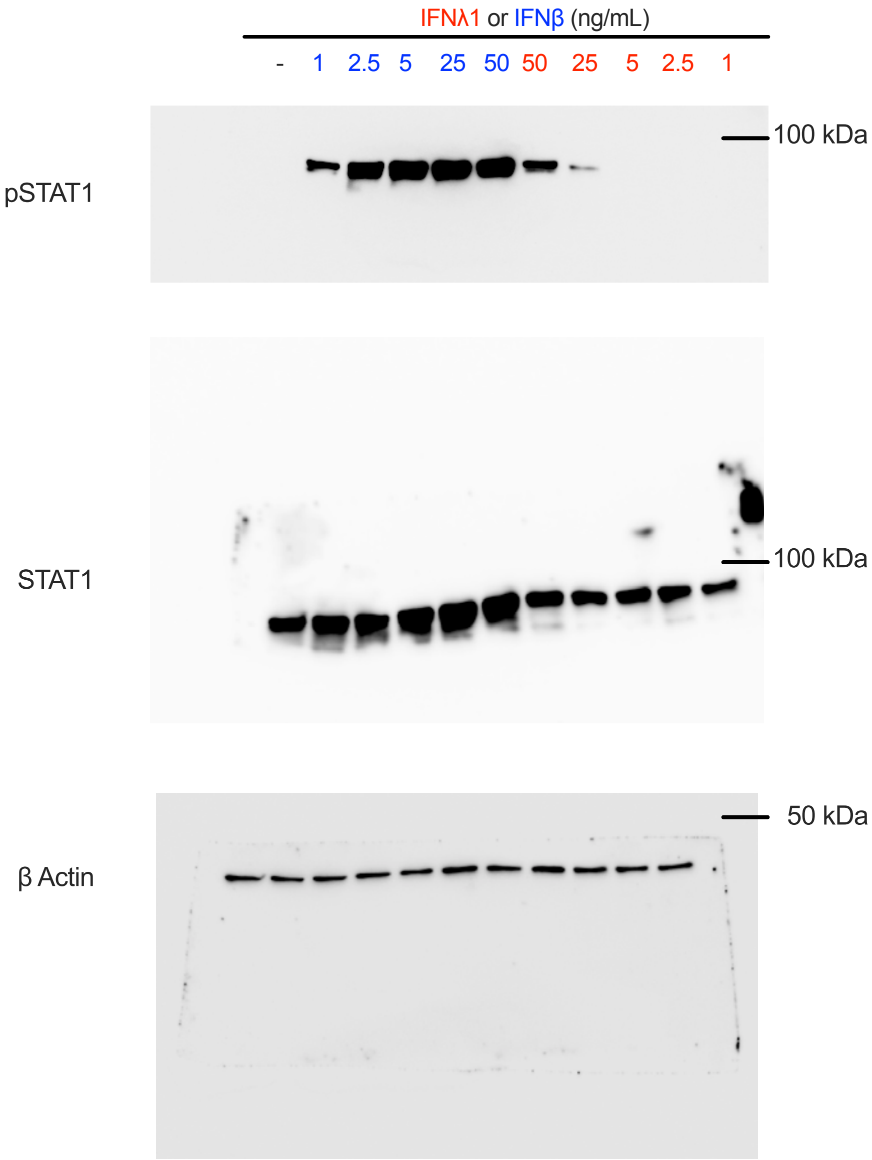

Figure 7C

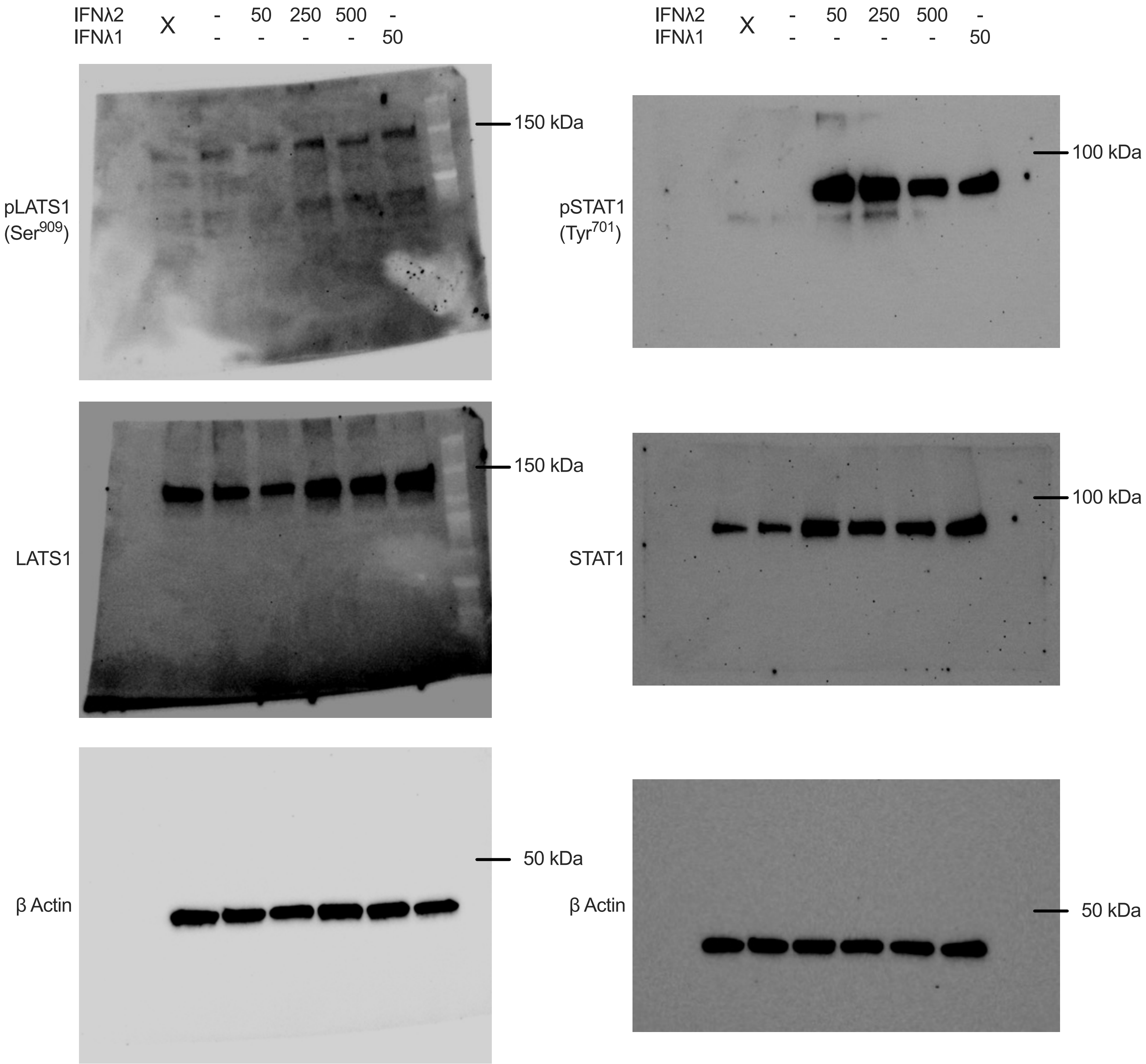
IFNλ2 X - 50 250 500 -
IFNλ1 - - - - 50
pLATS1 (Ser909)
150 kDa
LATS1
150 kDa
β Actin
50 kDa
IFNλ2 X - 50 250 500 -
IFNλ1 - - - - 50
pSTAT1 (Tyr701)
100 kDa
STAT1
100 kDa
β Actin
50 kDa

Figure S6C

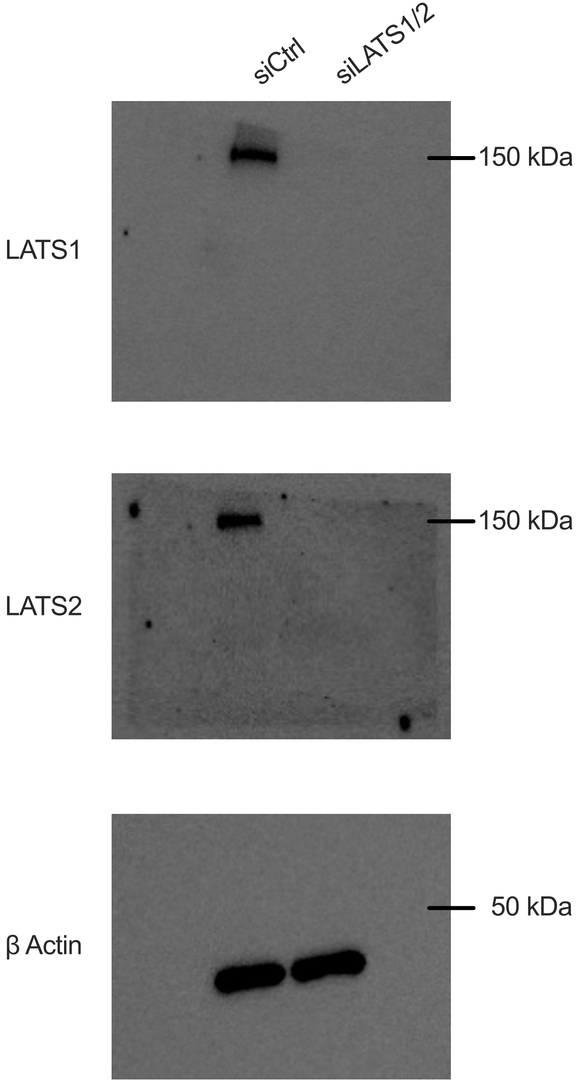

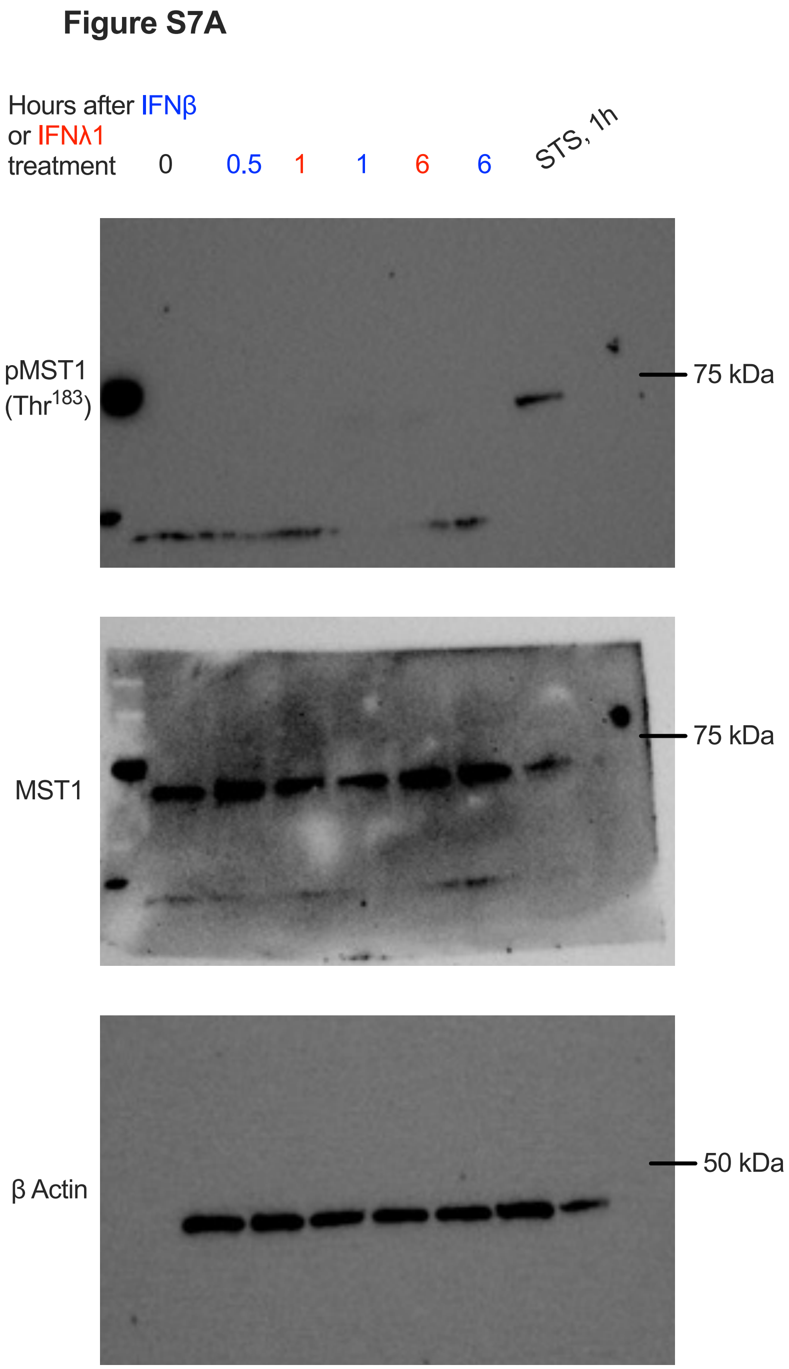
Figure S7A
Hours after IFNβ
or IFNλ1
treatment
0
0.5
1
1
6
6
STS, 1h
pMST1
(Thr183)
75 kDa
MST1
75 kDa
β Actin
50 kDa

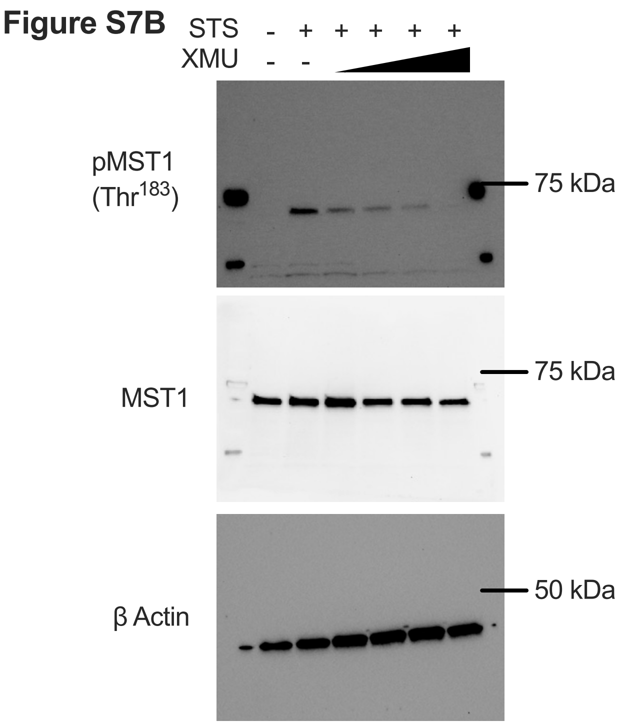
Figure S7B
STS - + + + + +
XMU - -
pMST1
(Thr183)
75 kDa
MST1
75 kDa
β Actin
50 kDa

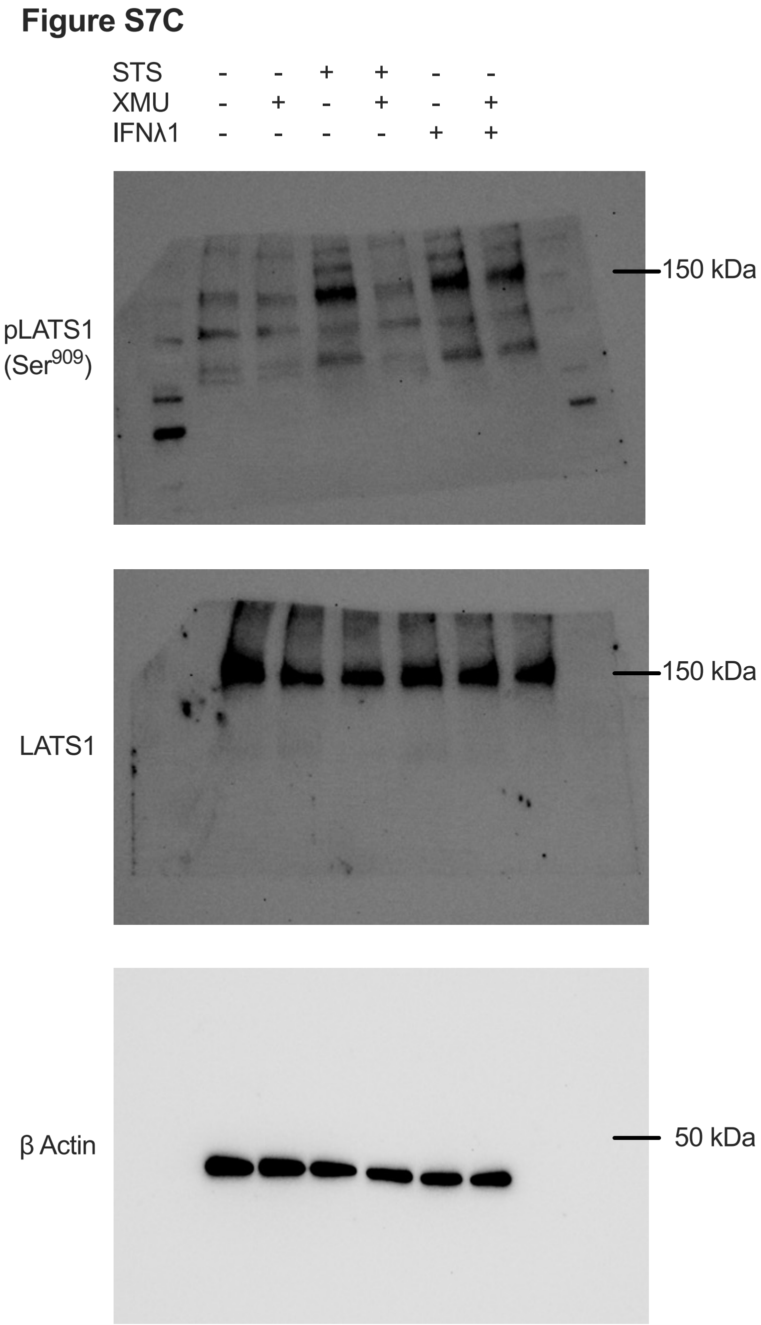
Figure S7C
STS - - + + - -
XMU - + - + - +
IFNλ1 - - - - + +
pLATS1
(Ser909)
150 kDa
LATS1
150 kDa
β Actin
50 kDa

Figure S12A

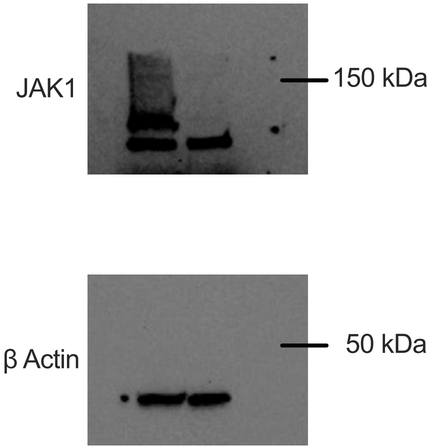

Figure S13A

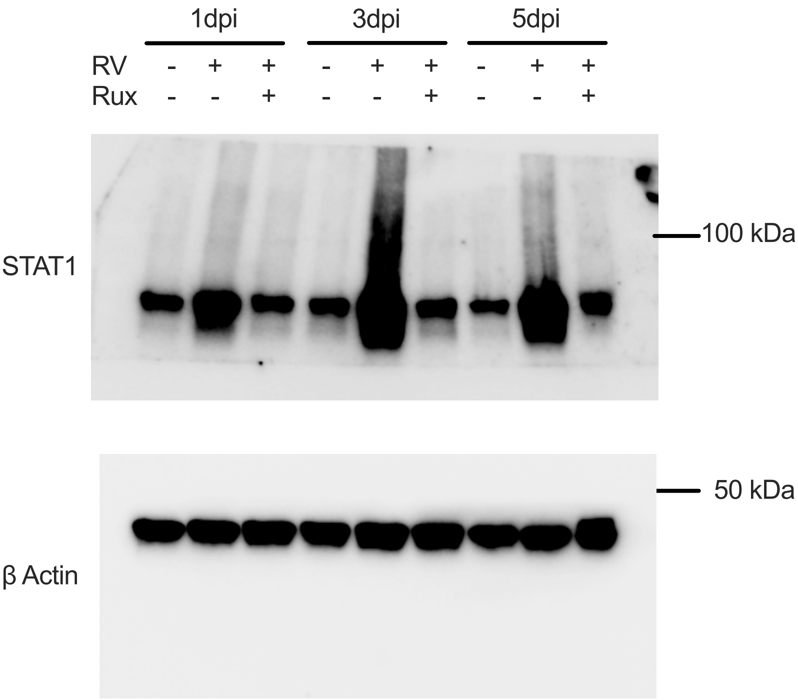

Figure S13B

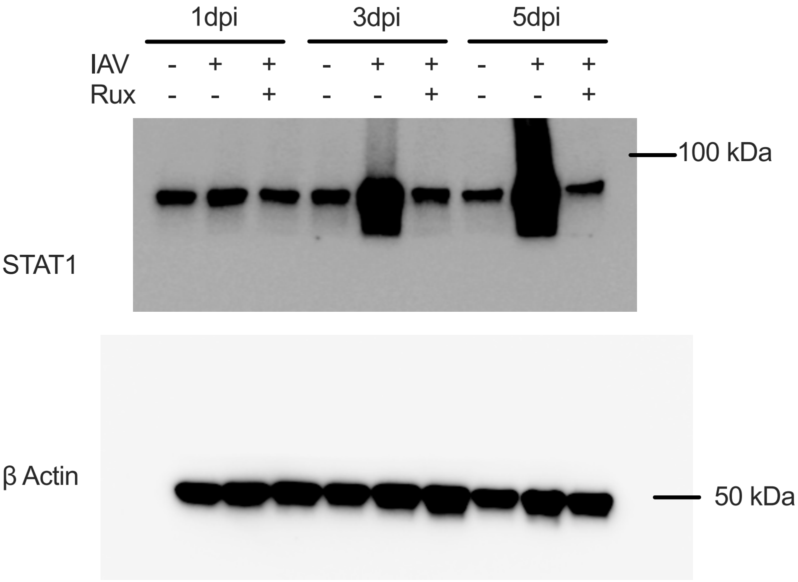

Supplement: S1 Raw Images — (PDF) [file pbio.3003615.s017.pdf]
